# Microbubble Shell Stiffness Engineering Enhances Ultrasound Imaging, Drug Delivery, and Sonoporation

**DOI:** 10.1002/adma.202507655

**Published:** 2025-11-17

**Authors:** Roman A. Barmin, MirJavad Moosavifar, Elena Rama, Julia Blöck, Anne Rix, Vladislav S. Petrovskii, Rustam A. Gumerov, Jens Köhler, Michael Pohl, Céline Bastard, Stephan Rütten, Laura Charlton, Vu Ngoc Khiêm, Fabio Domenici, Thomas Lisson, Ekaterina Savina, Rui Zhang, Jasmin Baier, Susanne Koletnik, Vasileios Koutsos, Mikhail Itskov, Gaio Paradossi, Georg Schmitz, Tina Vermonden, Laura De Laporte, Robert Göstl, Andreas Herrmann, Igor I Potemkin, Fabian Kiessling, Twan Lammers, Roger M. Pallares

**Affiliations:** ^1^ Institute for Experimental Molecular Imaging RWTH Aachen University Hospital 52074 Aachen Germany; ^2^ Institute for Bioengineering of Catalonia Barcelona 08028 Spain; ^3^ DWI – Leibniz Institute for Interactive Materials 52074 Aachen Germany; ^4^ Institute of Technical and Macromolecular Chemistry RWTH Aachen University 52074 Aachen Germany; ^5^ Department of Advanced Materials for Biomedicine Institute of Applied Medical Engineering RWTH Aachen University 52074 Aachen Germany; ^6^ Electron Microscope Facility RWTH Aachen University Hospital 52074 Aachen Germany; ^7^ School of Engineering Institute for Materials and Processes University of Edinburgh Edinburgh EH9 3FB UK; ^8^ Department of Continuum Mechanics RWTH Aachen University 52074 Aachen Germany; ^9^ Department of Chemical Science and Technologies University of Rome “Tor Vergata” Rome 00133 Italy; ^10^ Chair for Medical Engineering Faculty of Electrical Engineering and Information Technology Ruhr University Bochum 44801 Bochum Germany; ^11^ Division of Pharmaceutics Utrecht Institute for Pharmaceutical Sciences (UIPS) Utrecht University Utrecht TB 3508 The Netherlands; ^12^ Department of Chemistry and Biology University of Wuppertal 42119 Wuppertal Germany

**Keywords:** alkyl cyanoacrylate, drug delivery, microbubbles, sonoporation, ultrasound

## Abstract

Microbubbles (MB) are widely used as contrast agents for ultrasound (US) imaging and US‐enhanced drug delivery. While the majority of studies utilize commercial MB formulations, increasing experimental evidence indicates that distinct MB features critically determine their diagnostic and therapeutic performance. Here, it is shown that shell stiffness engineering of poly(alkyl cyanoacrylate) (PACA) MB, via introducing monomers with varying alkyl chain lengths and glass transition temperatures, preserves a narrow size distribution ≈2–3 µm, while enhancing MB drug loading, in vitro sonoporation capability, and in vitro and in vivo acoustic responses. All‐atom molecular dynamics simulations and spectroscopic experiments demonstrate that MB shell engineering increases drug diffusion rates in the shell, maximizing the loading capacity of the formulations. Atomic force microscopy demonstrates that the stiffness of the MB shell can be tailored by more than ten‐fold, boosting sonoporation and imaging performance. Altogether, the work provides new insights into the control of polymeric MB structure and performance via dedicated shell engineering, promoting applications in US imaging and therapy.

## Graphical abstract

Shell‐engineered poly(alkyl cyanoacrylate) microbubbles with tunable stiffness improve ultrasound imaging and therapy. Adjusting the length of the alkyl chain and the glass transition temperature maintains a narrow microbubble size distribution while enhancing drug loading, sonoporation and the acoustic response. Atomic force microscopy confirms over tenfold tunability in shell stiffness, enabling optimized imaging and therapeutic performance through precise polymer shell engineering.

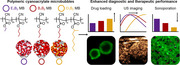

## 1 Introduction

Microbubbles (MB) are gas‐filled particles that are routinely used in the clinic for ultrasound (US) imaging and therapy.^[^ ^1^, ^2^ ^]^ Due to their size of 1–8 µm, MB are confined within blood vessels and do not extravasate into tissues. MB have been used as intravascular contrast agents, based on their ability to reflect US waves and provide better contrast than liquid or solid particles of the same size.^[^ ^2^ ^]^ Originally introduced into the clinic in the 1990s, MB are utilized to assess cardiac, hepatic, and renal lesions,^[^ ^3^, ^4^ ^]^ and first‐in‐human results of molecular US imaging with targeted MB in cancer patients were reported in 2017.^[^ ^5^, ^6^ ^]^ For drug delivery, US‐mediated MB oscillations induce a multitude of effects, including the generation of shear forces near vessel walls and the creation of local fluid flow patterns (e.g., microstreams), promoting the transient opening of biological barriers, such as vascular walls in tumors and the blood–brain barrier, by a mechanism known as sonoporation, thereby enhancing drug delivery and efficacy.^[^ ^7^ ^]^

The MB shell prevents rapid gas dissolution from its core.^[^ ^8^ ^]^ The shell furthermore dictates the MB oscillation capabilities, hence, MB acoustic response.^[^ ^8^, ^9^ ^]^ MB formulations have been traditionally divided into two major classes, namely soft‐ and hard‐shelled MB, based on their coating materials and stiffness profiles. Soft lipid (and to a lesser extent protein) shells are thin and promote greater US contrast signals than their hard polymeric counterparts.^[^ ^8^ ^]^ In contrast, polymeric shells can be loaded more efficiently with drugs, while still displaying adequate imaging capabilities, particularly when considering their compression‐dominated behavior and associated frequency shifts.^[^ ^10^, ^11^ ^]^ For instance, several polymeric MB formulations for diagnostic use have successfully completed (up to) Phase II clinical trials.^[^ ^12^, ^13^ ^]^ Moreover, the characteristics of polymeric MB, e.g., acoustic response and drug loading, can be manipulated by material selection and formulation design, for example, when the MB surface is conjugated with targeting agents for molecular imaging and targeted drug delivery.^[^ ^14^ ^]^

Among available shell materials, poly(butyl cyanoacrylate) (PBCA) is advantageous, because it provides MB with colloidal stability for months, adequate acoustic responses, and with a versatile functionalization toolbox available, while also being biocompatible and approved by the American Food and Drugs Administration as a surgical super‐glue.^[^ ^15^ ^]^ Previous studies have showcased the use of PBCA MB for molecular^[^ ^16^, ^17^ ^]^ and multimodal imaging,^[^ ^18^ ^]^ US‐mediated drug and gene delivery,^[^ ^10^, ^19^ ^]^ and blood‐brain barrier opening.^[^ ^20^, ^21^, ^22^ ^]^ However, hardly any efforts have been invested in improving the imaging and therapy performance of PBCA MB through varying the monomer composition and therewith enhancing the mechanical properties of the MB shell.^[^ ^23^, ^24^ ^]^ We have recently identified that synthetic manipulations and post‐functionalization can significantly enhance the acoustic responses of PBCA MB.^[^ ^14^, ^25^, ^26^, ^27^ ^]^ However, those approaches are limited by the inherent physicochemical features (e.g., shell thickness and stiffness) of the PBCA material.

**Figure 1:**
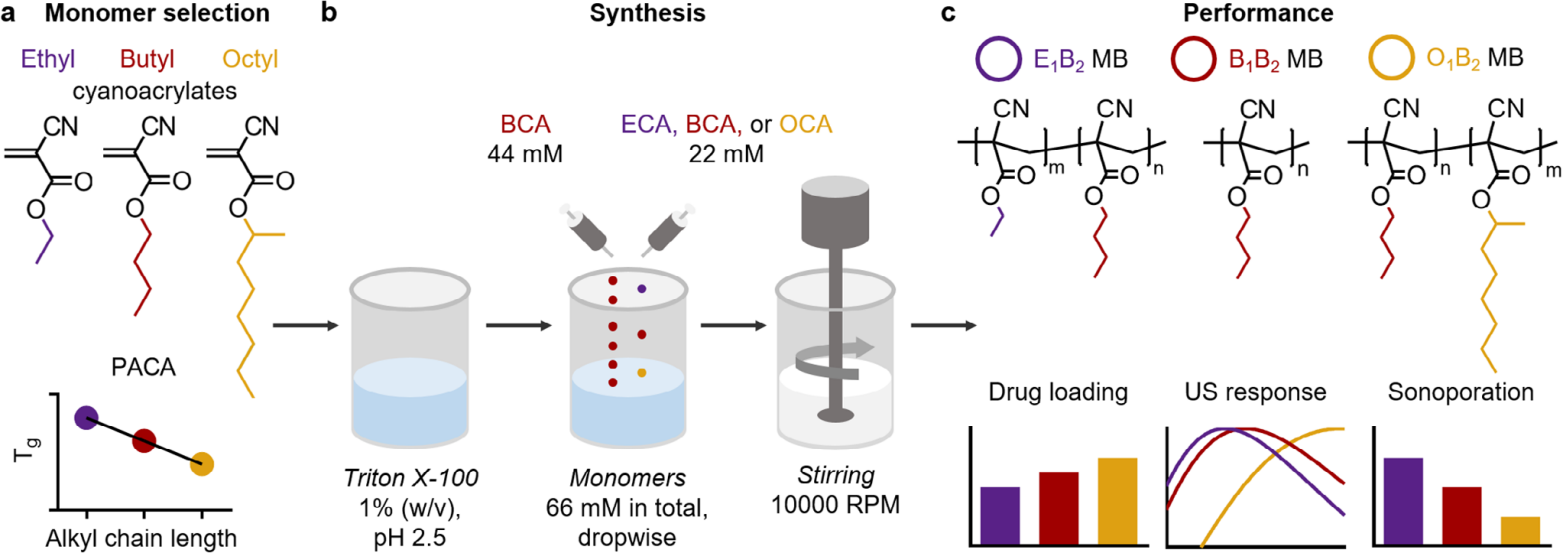
Experimental setup. a) Alkyl cyanoacrylate monomers with different alkyl side chain lengths were selected for PACA MB synthesis based on the trend of glass transition temperature (T_g_) reduction for the resulting polymer chains with longer alkyl side chains. b) Ethyl, butyl, or octyl cyanoacrylate monomers (ECA, BCA, or OCA, respectively) were mixed with a constant amount of BCA for anionic random copolymerization in the presence of Triton X‐100 under high‐speed stirring to produce MB. c) The resulting MB formulations were evaluated for drug loading, US response, and sonoporation performance.

Here, we present a new shell stiffness engineering approach that produces MB with customizable acoustic and drug‐loading features that go beyond those observed in conventional PBCA MB. This new method relies on the copolymerization of monomers that yield polymers with different MB shell stiffness profiles derived by atomic force microscopy (AFM). Three monomers were selected based on the trend of lower polymer rigidity (related to their glass transition temperatures, *T_g_*) with increasing alkyl side chain length (**Figure** **1**a). Ethyl, butyl, and octyl cyanoacrylate (ECA, BCA, and OCA, respectively) are commonly utilized monomers for surgical adhesives and nanomedicine fabrication, producing polymers with Tg of 132, 113, and 57 °C, respectively, as measured for polymer chains exceeding 100  kDa.^[^ ^28^ ^]^ Poly (alkyl cyanoacrylate) (PACA) MB were synthesized by randomly copolymerizing different alkyl cyanoacrylate monomers with BCA in the presence of Triton X‐100, which under stirring acted as an MB template for the polymer to grow on top, while keeping the total amount of monomers constant (Figure 1b). Since polymerization of ECA or OCA did not produce stable MB,^[^ ^29^ ^]^ BCA‐ECA and BCA‐OCA mixtures were copolymerized in a 2:1 molar ratio. MB samples are labelled throughout the manuscript to reflect the ratio of cyanoacrylate monomers introduced during the synthesis: E_1_B_2_ MB for the ECA‐based sample, B_1_B_2_ MB for the conventional PBCA MB previously reported by our group,^[^ ^10^, ^25^ ^]^ and O_1_B_2_ MB for the OCA‐based sample. After the synthesis, the polymeric MB were washed and stored in a 0.02 % (w/v) Triton X‐100 solution to avoid coalescence and aggregation, and were assessed for drug loading, acoustic response, and sonoporation capabilities. Together, our results demonstrate that tailoring the composition of polymeric MB enhances their performance for US imaging, sonoporation, and drug delivery applications (Figure 1c).

## 2 Results and Discussion

### 2.1 Cyanoacrylate Composition Maintains Narrow Diameter Distribution of Polymeric Microbubbles

**Figure 2:**
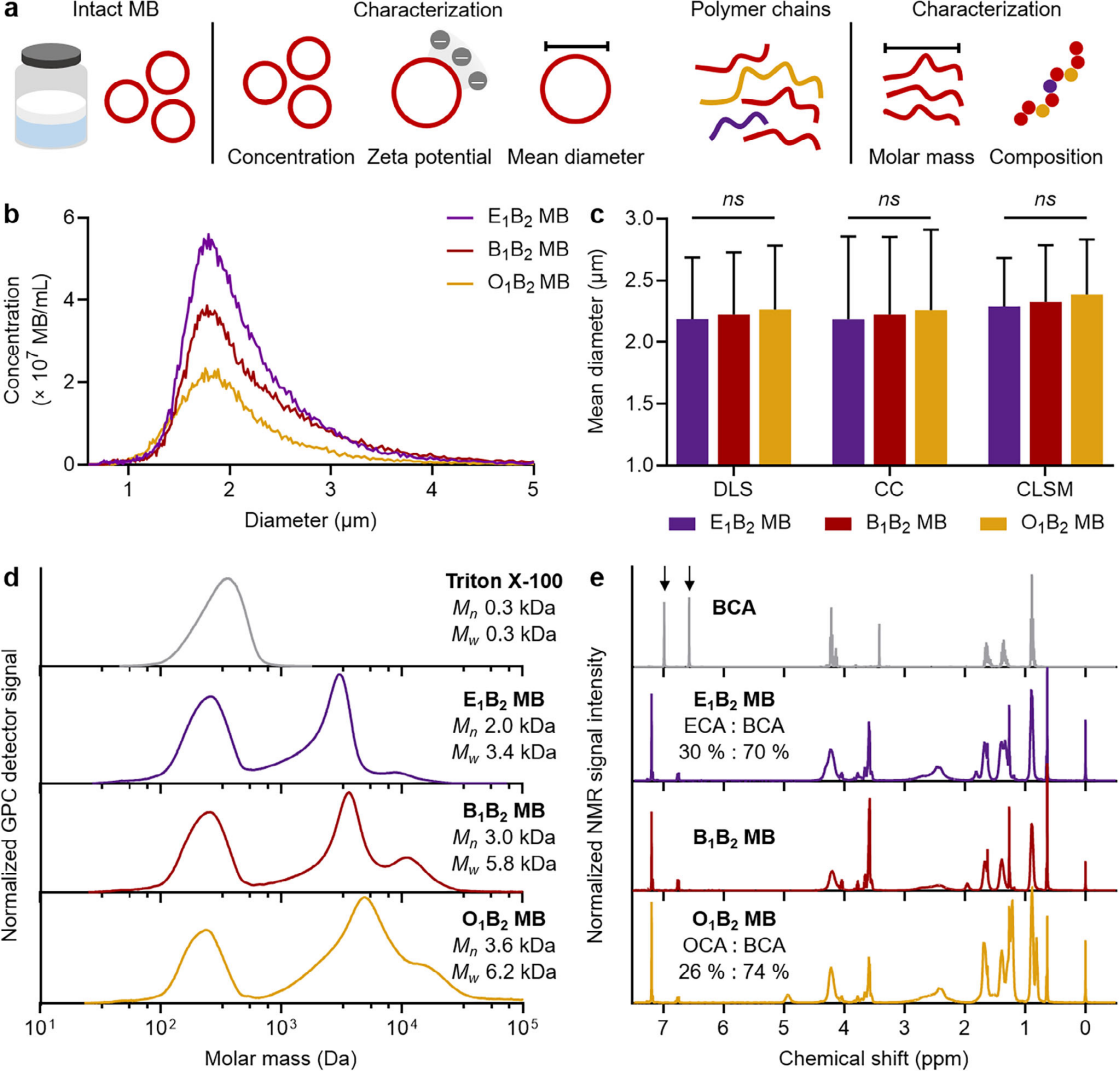
Cyanoacrylate composition tunes the physicochemical properties of MB. a) Schematic representation of the physicochemical characterization of intact MB and their polymer chains. b) Diameter distribution profiles, and c) mean diameter profiles obtained using different characterization techniques for the synthesized polymeric MB. d) Molar mass distribution profile from gel permeation chromatography (GPC), and e) proton nuclear magnetic resonance (NMR) spectra of polymeric MB, where arrows indicate the peaks originating from the monomeric BCA vinyl group. Values represent mean ± standard deviation of three different batches of polymeric MB, each measured in triplicates. (^*^) indicates groups that are significantly different with *p* < 0.05; (ns) indicates groups that are not significantly different with *p* > 0.05 (one‐way ANOVA with post hoc Tukey HSD test). Concentration, zeta potential, and polydispersity index values of MB are shown in Figure S1 (Supporting Information); NMR spectra of BCA monomer and Triton X‐100 with peak assignments are shown in Figure S2 (Supporting Information), and full peak assignments for NMR spectra of polymeric MB are provided in Figure S3 (Supporting Information).

The intact PACA MB and their polymer chains were analyzed as schematically shown in **Figure** **2**a. Figure 2b presents the diameter distribution of PACA MB, highlighting the absence of MB fraction smaller than 1 µm and showing that the majority fall within the 1–3 µm range. The presence of different cyanoacrylate monomers significantly affected the final concentration of the synthesized MB batches (Figure S1a, Supporting Information), as E_1_B_2_ MB had a 38.3 ± 11.5 % higher concentration compared to B_1_B_2_ MB, while O_1_B_2_ MB had a 30.2 ± 6.5 % lower concentration than B_1_B_2_ MB. The longer alkyl chain length of the monomer diminishes its reactivity by introducing steric hindrance,^[^ ^28^, ^30^ ^]^ which may impede the polymerization process and the resulting MB yield. The introduction of cyanoacrylate monomers, however, did not have statistically significant effects on the zeta potential and diameter distribution of the synthesized MB. The zeta potential values were measured as indicators of MB interfacial charge, and the E_1_B_2_ MB and B_1_B_2_ MB samples showed values of −27.4 mV, whereas the O_1_B_2_ MB sample exhibited a slightly lower value of ‐23.0 mV (Figure S1b, Supporting Information). This shift could be attributed to the greater steric hindrance and charge shielding on the MB surface by the longer OCA side chains compared to the shorter ECA and BCA counterparts. The mean diameter distributions of all MB samples were in the range of 2.2 ± 0.6 µm as confirmed by dynamic light scattering (DLS), Coulter Counter (CC) measurements of plain MB, and confocal laser scanning microscopy (CLSM) images of fluorescent dye (coumarin 6) loaded MB (Figure 2c). In addition, polydispersity index values obtained from DLS measurements were below 0.2, indicating low polydispersity (Figure S1c, Supporting Information).

To gain further insight into the composition of the polymeric MB, gel permeation chromatography (GPC) and proton nuclear magnetic resonance (NMR) spectroscopy were conducted. For the GPC and NMR analyses, the polymeric MB were washed against DI water to remove free Triton X‐100 from the samples, freeze‐dried, and the resulting powders were dissolved in chloroform. The GPC molar mass distribution profiles indicated that the polymeric MB were composed of polymer chains with number average molar mass (*M_n_*) and weight average molar mass (*M_w_*) values below 40 kDa (Figure 2d), which may facilitate their faster hydrolytic degradation compared to their longer counterparts once the MB are taken up by the liver and spleen.^[^ ^28^, ^31^ ^]^ The signal at ≈300 Da indicated the presence of Triton X‐100 residues in the MB shells and was comparable to that observed in the Triton X‐100 solution alone. The surfactant bubbles serve as colloidal templates during the MB formation, suggesting that the polymeric MB are likely to contain Triton X‐100. Furthermore, the GPC chromatograms for the MB samples exhibited a bimodal molecular weight distribution, with the primary band occurring between 3 and 5 kDa and secondary bands emerging between 8 and 12 kDa. The introduction of monomers with longer alkyl chains gradually increased the *M_n_* and *M_w_* values of the resulting polymer chains. This observation agrees with previous reports on anionic emulsion copolymerization of PACA chains and nanoparticles.^[^ ^32^, ^33^ ^]^ Hence, we hypothesize that the increased hydrophobicity of cyanoacrylate monomers with longer side chains facilitates higher molar masses of the resulting PACA chains entangled within MB shells.

The NMR spectra of the samples demonstrated that the polymeric MB shells were composed of PACA chains, as illustrated in Figure 2e. The BCA polymerization could be confirmed by the absence of peaks originating from the monomeric cyanoacrylate vinyl group (at 6.57 and 6.99 ppm), as described in the literature^[^ ^34^ ^]^ (complete peak assignment for BCA monomer is provided in Figure S2a, Supporting Information). The presence of Triton X‐100 residues was confirmed by the presence of peaks originating from the surfactant (shown in Figure S2b, Supporting Information) in the spectra of the PACA chains (complete peak assignments are provided in Figure S3, Supporting Information). According to the NMR analysis, Triton X‐100 residues accounted for 4 %, 7 %, and 3 % of the signal of E_1_B_2_ MB, B_1_B_2_ MB, and O_1_B_2_ MB, respectively, which agreed with the GPC data (a detailed explanation of the quantification procedure is provided in the Experimental section). The presence of peaks originating from the ECA and BCA monomers confirmed their copolymerization within the E_1_B_2_ MB shells, and the 30 % proportion of ECA units within the E_1_B_2_ MB shells was close to the initial monomer ratios introduced during synthesis (as quantified based on selected signal intensities, as described in the Experimental section). Conversely, the proportion of OCA units within the chains of O_1_B_2_ MB was 26 %. We hypothesize that this lower value results from the reduced reactivity and greater steric hindrance of the OCA monomer compared to its BCA and ECA counterparts. The combined results of the GPC and NMR analyses demonstrated the presence of copolymers made of the different cyanoacrylates introduced during the MB synthesis (i.e., ECA, BCA, and OCA) within the shells.

### 2.2 Cyanoacrylate Composition Finetunes the Shell Stiffness of Polymeric Microbubbles

**Figure 3:**
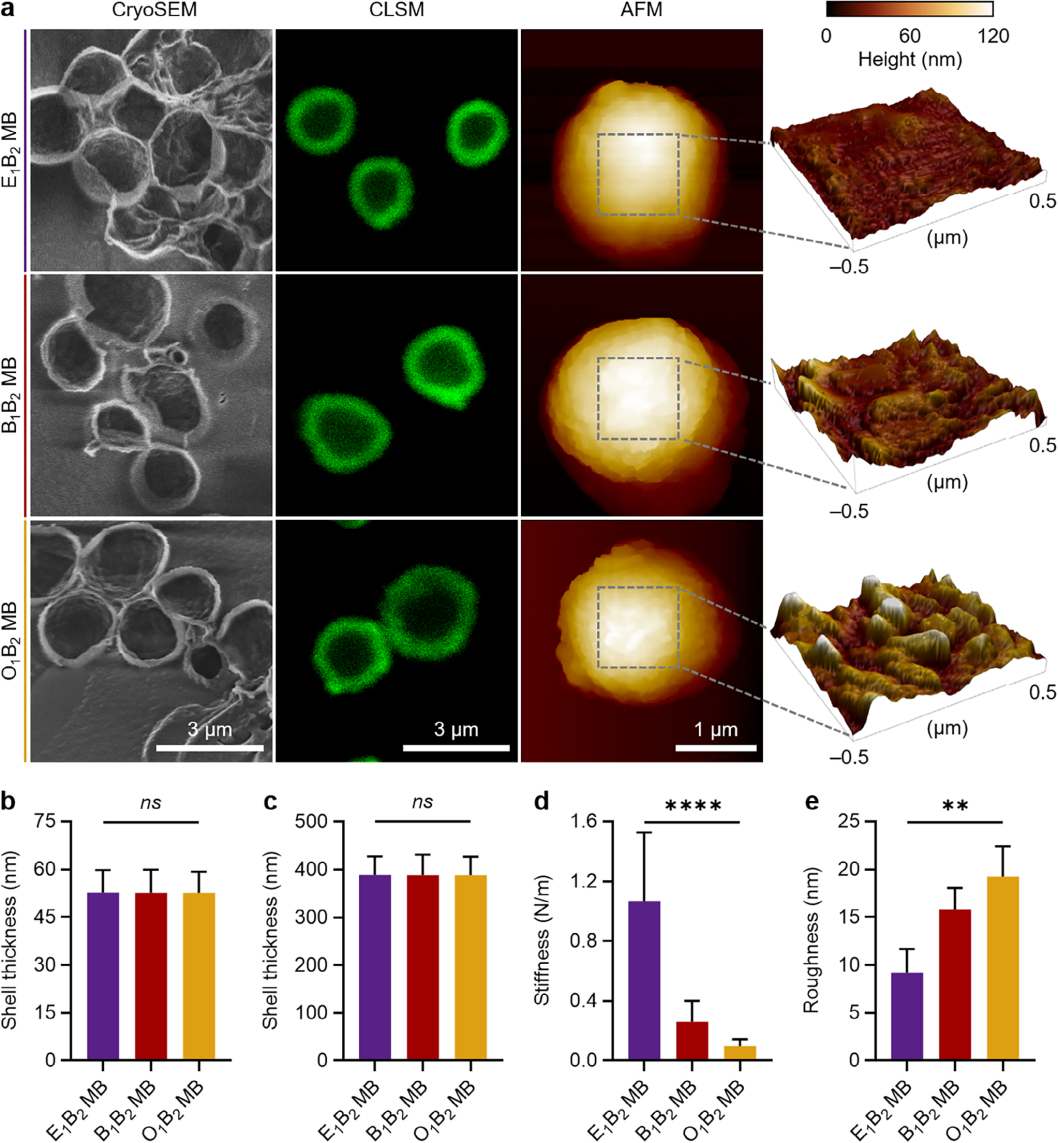
Morphology and stiffness of shell‐engineered PACA MB. a) Representative cryoSEM, CLSM, and AFM micrographs of MB. The surface topographies of 1 × 1 µm MB shell sections are obtained from the AFM micrographs. Shell thickness values of the PACA MB measured by b) cryoSEM and c) CLSM. d) Stiffness and e) surface roughness values derived as root mean square values of the MB surface measured by AFM. (^**^) and (^****^) indicate groups that are significantly different with *p* < 0.01 and *p* < 0.0001, respectively, and (ns) indicates groups that are not significantly different with *p* > 0.05 (one‐way ANOVA with post hoc Tukey HSD test). Representative wide‐area cryoSEM and CLSM micrographs of MB samples are shown in Figure S4 (Supporting Information), individual data points for MB shell thickness and AFM‐derived stiffness measurements are presented in Figure S5 (Supporting Information), and representative optical micrographs of individual MB under gradually increasing force applied by a cantilever are shown in Figure S6 (Supporting Information).

Subsequently, plain MB and MB loaded with the fluorescent dye were imaged by scanning electron cryo‐microscopy (cryoSEM) and CLSM, respectively. The copolymerization of different cyanoacrylate monomers within the MB shells did not affect the MB morphology observed by cryoSEM and CLSM. Representative images are shown in **Figure** **3**a, and wide‐area micrographs are presented in Figure S4 (Supporting Information). All MB samples exhibited shell thickness values of 50 nm according to cryoSEM micrographs (Figure 3b), and 400 nm according to CLSM micrographs (Figure 3c), which is consistent with our previous reports on PBCA MB.^[^ ^25^, ^26^ ^]^ The discrepancy in shell thickness values across methods is likely due to the MB shrinkage during the freezing process required for cryoSEM, while high‐quality CLSM microscopy is anticipated to yield a more accurate depiction of shell thickness, as MB are maintained in an intact state within a liquid environment.

The stiffness values of the MB shells followed the *T_g_* of the PACA according to the AFM results (Figure 3d). To conduct these measurements, an AFM cantilever was employed to exert force on individual MB that were attached to a substrate and preserved in a liquid environment. The force necessary to deform the MB was measured to calculate the stiffness of the MB shells. Single data points obtained for shell thickness and stiffness measurements are shown in Figure S5 (Supporting Information). While E_1_E_2_ MB had the stiffest shell among the samples, all three samples showed a decreasing MB shell stiffness trend with increasing MB diameter (Figure S5c, Supporting Information), consistent with previous reports on microspheres of different compositions, as the (AFM‐derived) stiffness of spherical shells with constant wall thickness decreases with the increasing diameter.^[^ ^35^, ^36^ ^]^ Notably, the majority of the MB population was centred ≈2.2 µm in diameter (Figure 2b), with only a small fraction exceeding 3 µm, indicating that the observed stiffness trends remain representative for the majority of the MB populations. Figure S6 (Supporting Information) shows representative optical micrographs of individual MB from each sample under progressively increasing force applied by a cantilever until MB breakage: while O_1_B_2_ MB were destroyed under a force of 50 nN, E_1_B_2_ MB could withstand up to 190 nN.

The cyanoacrylate composition also affected the roughness of the MB shell, as shown in representative AFM micrographs in Figure 3a and quantified in Figure 3e. For instance, O_1_B_2_ MB had a surface roughness value (derived as a root mean square value) of 19.2 ± 2.6 nm, compared to the values of 15.8 ± 2.8 and 9.2 ± 2.0 nm for the B_1_B_2_ MB and E_1_B_2_ MB counterparts, respectively. We hypothesize that longer side chains in cyanoacrylate monomers may increase surface roughness due to increased entanglement between the resulting polymer chains.

Therefore, these results highlight that tuning the cyanoacrylate composition of the polymeric PACA MB does not change the mean MB diameter, surface charge, and shell thickness, while it affects MB concentration, molecular weight of polymer chains, and more importantly, provides PACA MB with tunable (AFM‐derived) shell stiffness.

### 2.3 Cyanoacrylate Composition Improves Drug Loading in Polymeric Microbubbles

**Figure 4:**
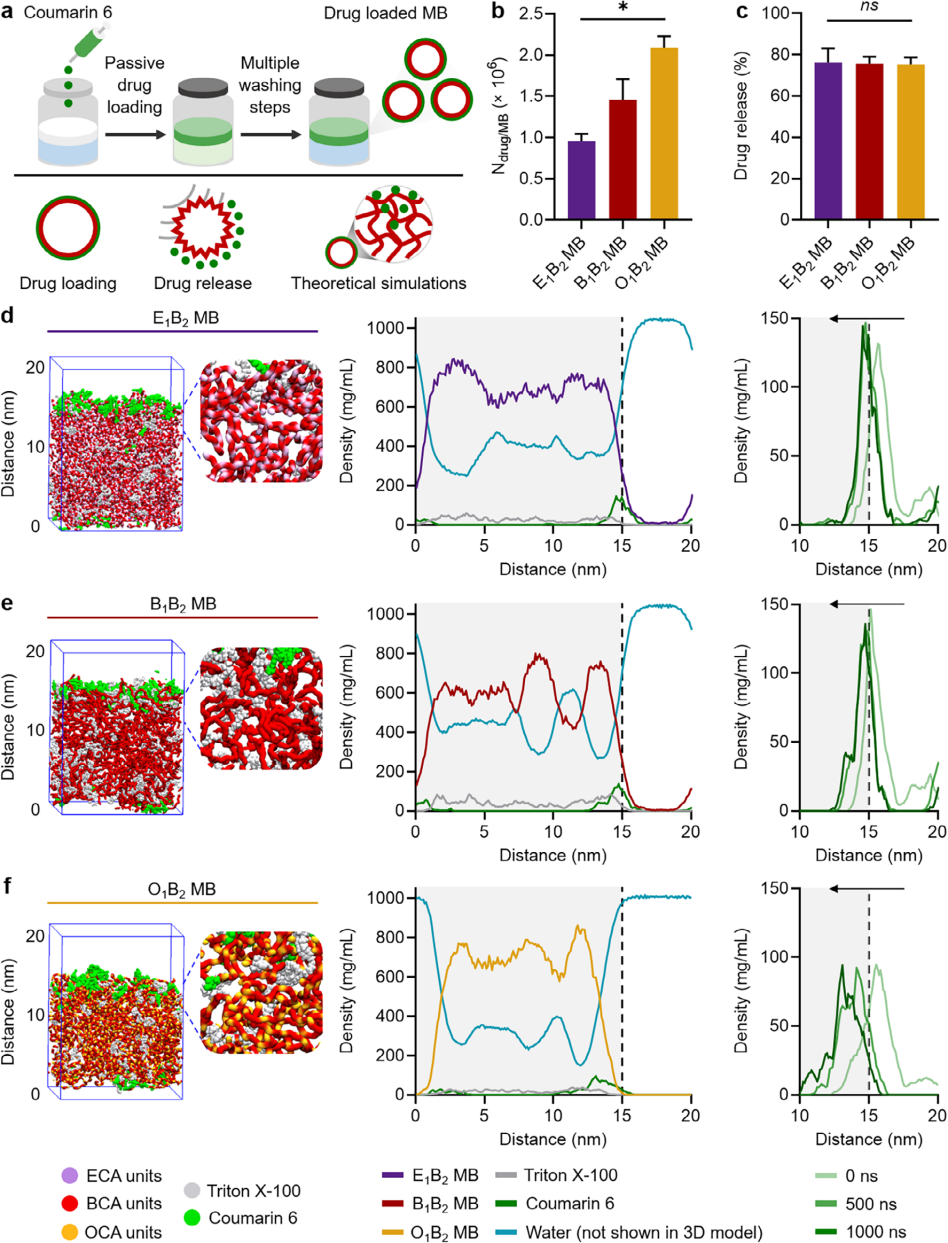
Drug loading capabilities of PACA MB. a) Schematic representation of the coumarin 6 loading protocol and the experimental and simulation workflow of the study. b) Numbers of coumarin 6 molecules loaded per MB (N_drug/MB_) and c) drug release percentage by the different MB samples upon US exposure. d–f) Simulation snapshots of the MB shells near the water‐shell interface upon coumarin 6 loading, corresponding density profiles of the MB shells synthesized with different cyanoacrylate compositions, and coumarin 6 diffusion profiles represented by the density profiles at three equal time points during one microsecond of the simulation study for (d) E_1_B_2_ MB, (e) B_1_B_2_ MB, and (f) O_1_B_2_ MB, respectively. The gray background in the density profiles represents the MB shell, and the dashed lines correspond to the MB shell‐liquid interface. N_drug/MB_ and drug release values represent mean ± standard deviation of three different batches of drug‐loaded MB, measured in triplicates. (^*^) indicates groups that are significantly different with *p* < 0.05, (ns) indicates groups that are not significantly different with *p* > 0.05 (one‐way ANOVA with post hoc Tukey HSD test). In the simulation snapshots, the water molecules are not displayed for clarity, however, they were considered during the simulation, as shown in the density profiles.

Since the shell properties determine the drug delivery performance of MB, we investigated the drug loading and release capacities of the different polymeric MB, as schematically presented in **Figure** **4**a. Coumarin 6 was selected as a drug model because of its strong fluorescence emission and hydrophobicity (*logP* value of 4.9), which is similar to clinical anticancer drugs, such as cabozantinib, lapatinib, and cabazitaxel.^[^ ^37^ ^]^ Coumarin 6 was loaded into the MB shells after synthesis according to a previously established protocol that exploits the hydrophobic interactions between the drug and the polymer chains, and washing steps were implemented to remove unloaded molecules from drug‐loaded MB (Figure 4a).^[^ ^38^ ^]^ As shown in Figure 4b, the number of coumarin 6 molecules loaded in each sample increased with the introduction of longer alkyl chain monomers. For instance, while standard B_1_B_2_ MB encapsulated 1.45 × 10^6^ drug molecules per MB (equivalent of 85 ± 12 ng per 1 × 10^9^ MB, as listed in Table S1, Supporting Information), the O_1_B_2_ MB contained 1.4‐fold more drug molecules per MB. We hypothesize that this is due to increased hydrophobicity provided by the longer side chains of the OCA units compared to the BCA counterparts, and the greater MB shell roughness of O_1_B_2_ MB compared to B_1_B_2_ MB. Drug release values upon US exposure of all samples, however, were in the same range, between 60 and 80% (Figure 4c), and were consistent with our previous reports.^[^ ^18^ ^]^ The hydrophobic interactions allow the release of entrapped drug molecules from the polymeric matrix, especially during US‐mediated MB destruction.^[^ ^39^ ^]^ Our team has previously studied drug entrapment during and after MB synthesis and found the release rates in both methods comparable to those reported in this study.^[^ ^10^ ^]^ Therefore, polymeric MB synthesized using cyanoacrylate monomers with a longer alkyl side chain could carry higher amounts of drug molecules than standard PBCA MB, while releasing their payload with the same efficiency under US pulses.

To understand the factors influencing coumarin 6 loading efficiency by tuning the cyanoacrylate composition of the MB shells, we performed an all‐atom molecular dynamics simulation. We simulated three systems: E_1_B_2_ MB, B_1_B_2_ MB, and O_1_B_2_ MB based on the GPC and NMR experimental results presented above (namely identical monomer ratios, numbers of monomers within the chains, and Triton X‐100 contents within the MB shells). Each system, simulated in a box of 15 nm alongside each axis, was filled with the corresponding polymer chains and Triton X‐100, while water molecules filled the remaining cavities. It is important to note that the modeled section is significantly thinner than the entire MB shell and represents a section near the interface with the aqueous medium. The final composition of the simulation boxes is shown in Table S2 (Supporting Information), and representative simulation snapshots are shown in Figures S6a–c (Supporting Information), for E_1_B_2_ MB, B_1_B_2_ MB, and O_1_B_2_ MB, respectively. The polymer and aqueous phase did not mix but formed a network of nanopores or nanocavities (Figure S7, Supporting Information), consistent with previous dissipative particle dynamic simulations of PBCA MB shells.^[^ ^26^ ^]^ Triton X‐100 molecules, being amphiphilic, positioned themselves at the interface of the pores, with the hydrophobic part adsorbed to the polymer phase and the hydrophilic tail extending into the water. Triton X‐100 was uniformly distributed throughout the volume of each simulated system.

For the drug loading simulation, we expanded the simulation box in the z‐direction by 5 nm and placed coumarin 6 molecules in the resulting space near the bubble boundary (Figure 4d–f for E_1_B_2_ MB, B_1_B_2_ MB, and O_1_B_2_ MB, respectively). Subsequently, the diffusion of coumarin 6 was simulated over a period of one microsecond, representing the practical upper limit of current computational resources and allowing the system to reach equilibrium.^[^ ^40^, ^41^ ^]^ Diffusion coefficients of 1.4 ± 0.6, 1.5 ± 0.6, and 2.7 ± 0.3 µm^2^ s^−1^ were obtained for the model drug molecules in E_1_B_2_ MB, B_1_B_2_ MB, and O_1_B_2_ MB, respectively, highlighting a gradual increase in drug molecule diffusion from E_1_B_2_ MB to O_1_B_2_ MB samples. Corresponding density profiles were constructed to analyze the dynamics of coumarin 6 penetration (Figure 4d–f), showing that the model drug was mainly located at the interface between water and the polymer matrix of MB shells and slowly diffused into the polymer matrix for all three systems. A more detailed analysis of the coumarin 6 density profile along the z‐axis at different time points (initial, middle, and end of the simulation) shows that the density band shifted toward the polymer region in all cases, with the largest shift observed for the O_1_B_2_ MB, due to the more flexible structure and increased local hydrophobicity of this system compared to its counterparts. After one microsecond of the simulation study, the cumulative density under the curve of coumarin 6 within the polymer matrix (taken as the distance between 0 and 15 nm) was 121.7, 137.4, and 193.8 mg × nm mL^−1^ for E_1_B_2_ MB, B_1_B_2_ MB, and O_1_B_2_ MB, respectively, which is consistent with the experimental results of drug loading in PACA MB (shown in Figure 4b).

Therefore, the simulations confirmed the experimentally observed drug loading trend, and provided further mechanistic insights. Introducing monomers with longer alkyl chains into the MB shell promoted greater local hydrophobicity and roughness, facilitating hydrophobic drug loading into the MB shell matrix.

### 2.4 Cyanoacrylate Composition Amplifies Microbubble Acoustic Responses

**Figure 5:**
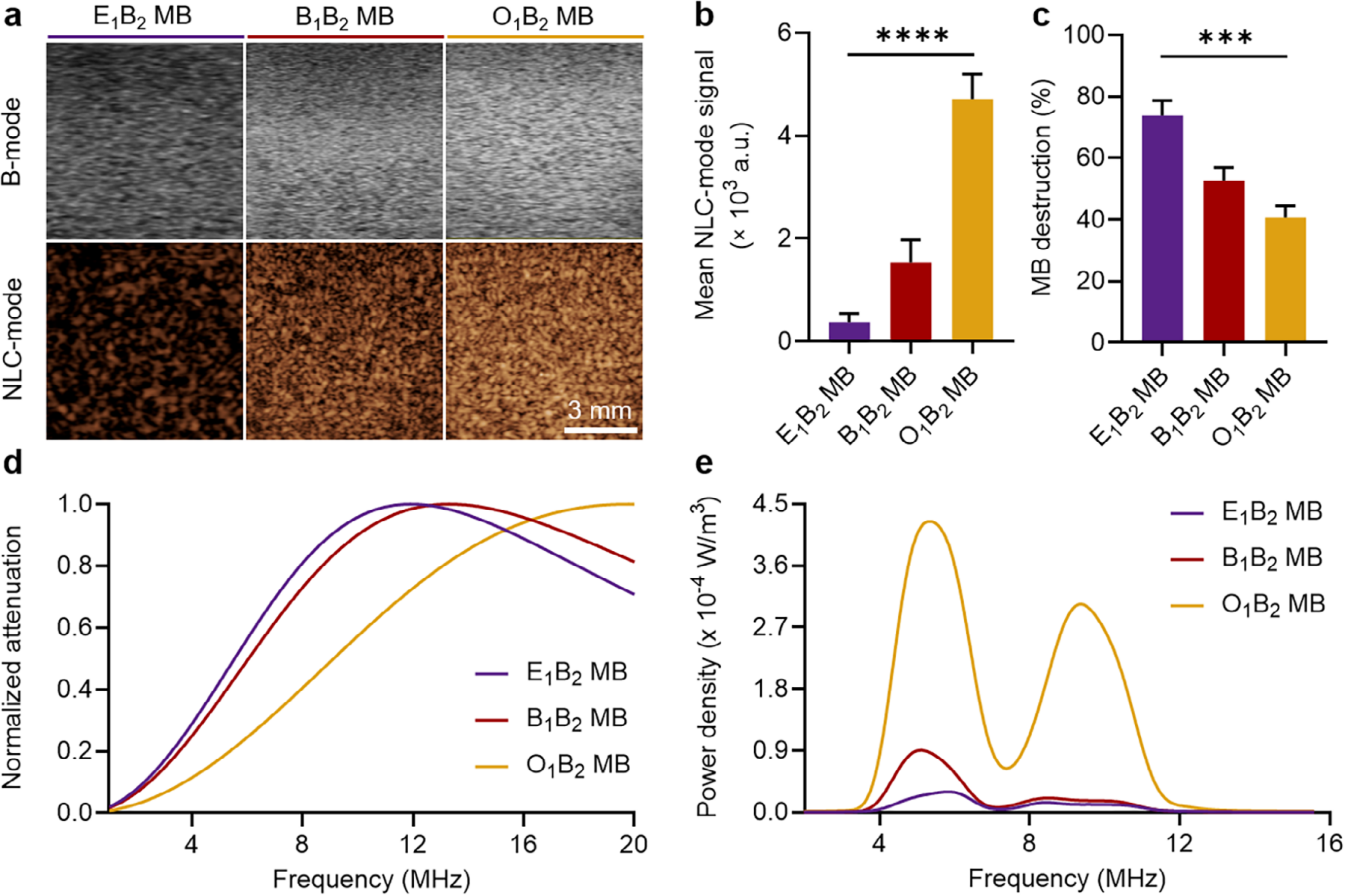
Acoustic properties of shell‐optimized PACA MB. a) Representative B‐ and NLC‐ mode sonograms of the polymeric MB at 4 % power and 18 MHz central frequency (scale bar: 3 mm). b) Quantified mean signal intensities of the polymeric MB at 4 % in NLC mode (arbitrary units, a.u.). c) PACA MB destruction rate after exposure to 10 % power for 5 s. d) Normalized attenuation spectra of the PACA MB recorded in the range of 0.5 – 20 MHz after three repeatable measurements at each measured concentration. e) Backscattering spectra of the PACA MB recorded in the range of 4.03–12.05 MHz after MB excitation with a short pulse at a center frequency of 5 MHz and acoustic pressure of 635 kPa. (^***^) and (^****^) indicate groups that are significantly different with *p* < 0.001 and *p* < 0.0001, respectively (one‐way ANOVA with post hoc Tukey HSD test). Quantified mean signal intensities of the polymeric MB at 4 % in B‐mode are shown in Figure S8 (Supporting Information), experimental and simulated destruction rates of the PACA MB at different acoustic powers are shown in Figure S10 (Supporting Information), acoustic attenuation spectra of the MB at different concentrations together with theoretically estimated shell viscosities and attenuation‐derived stiffness values are presented in Figure S12 (Supporting Information).

To comprehensively assess MB acoustic behavior, we characterized their responses using a preclinical US imaging setup with a central frequency of 18 MHz, attenuation measurements spanning 0.5–20 MHz, and backscattering measurements in the 3 – 12 MHz frequency range. Initially, we characterized the acoustic performance of the synthesized MB in both brightness mode (B‐mode; related to the intensity of the US signal reflected by the sample) and non‐linear contrast mode (NLC‐mode; designed to reflect MB oscillations and followed a pulse amplitude modulation scheme) at 4% power in a preclinical setup with a central transducer frequency of 18 MHz, which is commonly used in small animal in vivo imaging. All samples were highly responsive in both modes (**Figure** **5**a). Under 18 MHz frequency, the softer O_1_B_2_ MB showed a 12.6‐fold increase in NLC signal compared to their more rigid counterparts, E_1_B_2_ MB, and a 3.1‐fold increase in NLC signal compared to conventional B_1_B_2_ MB (Figure 5b). A similar trend of signal intensity increase was observed in B‐mode (Figure S8, Supporting Information), consistent with our previous reports.^[^ ^21^, ^25^ ^]^ Because MB diameter and shell thickness aspects were not statistically different in all samples, we hypothesize that modifications to the (AFM‐derived) shell stiffness (described in Figure 3d) was the main factor contributing to a greater MB oscillation upon exposure to US and, therefore, a higher contrast signal.

To simulate a prolonged US imaging scenario, MB acoustic stability was assessed over 5 min using a flow phantom under a central frequency of 18 MHz. SonoVue and Optison were evaluated as clinical‐grade soft‐ and hard‐shelled MB, respectively. B‐mode and NLC‐mode imaging (Figure S9a,c, Supporting Information) demonstrated sustained signal from polymeric MB under 4% acoustic power, with signal intensity increasing from E_1_B_2_ MB to O_1_B_2_ MB. SonoVue exhibited lower – yet persistent – signals consistent with previous findings, since it operates better at frequencies around (or below) 5 MHz.^[^ ^21^ ^]^ Optison initially produced signals comparable to O_1_B_2_ MB but showed rapid decay, falling below E_1_B_2_ MB and B_1_B_2_ MB after 3.5 min, likely due to its limited mechanical stability.^[^ ^42^, ^43^ ^]^ Mean signal intensity analysis (Figure S9b,d, Supporting Information) confirmed that O_1_B_2_ MB generated the strongest B‐mode and NLC‐mode signals from all the formulations tested.

We determined the percentage of MB destruction after relatively higher‐power US exposures using the same setup. MB with increased (AFM‐derived) stiffness showed greater destruction after US exposures of 10, 15, and 25% power (Figure 5c; Figure S10a,b, Supporting Information, respectively). This observation is consistent with previous reports showing that softer MB tend to better withstand acoustic pressures before rupturing, which can reduce the likelihood of immediate bursting under high‐power US exposure.^[^ ^44^, ^45^ ^]^ When the acoustic power was as high as 100%, all MB were completely destroyed (Figure S10c, Supporting Information). The MB destruction rate was also estimated using the void nucleation‐damage theory (Figure S11, Supporting Information),^[^ ^46^ ^]^ where each MB system is modeled by a representative cumulative polymer chain whose number of Kuhn segments are extracted from the MB shell characteristics, including (AFM‐derived) stiffness, thickness and polymeric molecular weight. The predicted destruction ratios in the three MB systems are shown in Figure S10c (Supporting Information) and are comparable to the experimental data described above. As E_1_B_2_ MB are the stiffest among the described systems, their representative cumulative chain is the shortest (i.e., with an estimated number of Kuhn segments of 21.2), so its limit of chain extensibility is reached earlier, resulting in a higher destruction ratio of the sample. Conversely, O_1_B_2_ MB are the softest with an estimated cumulative number of Kuhn segments of 4297.5, and their chains reach the limit of chain extensibility much later, resulting in lower MB destruction ratio.

To better understand the acoustic response of the MB samples at different US frequencies, we performed attenuation measurements, which provide insight into the absorption and scattering of acoustic energy by the MB exposed to an acoustic field.^[^ ^47^, ^48^ ^]^ We observed the gradual shift of the maximum attenuation response frequencies from 11.9 MHz to 13.3 MHz, and to 19.8 MHz (Figure 5d; Figure S12a–c, Supporting Information for each individual MB system) with reduced shell stiffness as measured by AFM (E_1_B_2_ MB, B_1_B_2_ MB, and O_1_B_2_ MB, respectively). Therefore, the maximal attenuation frequency shift correlates well with the NLC intensity signals observed in Figure 5a. All MB showed good echogenicity with maximum attenuation values increasing linearly with MB concentration at the corresponding (maximal) frequencies, as analyzed in Figure S12d (Supporting Information), and E_1_B_2_ MB exhibited higher maximum attenuation values compared to B_1_B_2_ MB and O_1_B_2_ MB counterparts at comparable MB concentrations. Based on the collected attenuation data, we performed theoretical estimations of shell stiffness and viscosity values (according to^[^ ^49^, ^50^ ^]^), with the resulting values presented in Figure S12e (Supporting Information). The attenuation‐estimated shell stiffness values exhibited an opposite trend compared to the ones obtained from AFM data shown in Figures 3d and S5c (Supporting Information). We hypothesize that the observed discrepancy arises from the fundamentally different deformation modes probed by the two stiffness measurements. AFM measures 1D, low‐frequency (sub‐MHz) local indentation of the shell, primarily reporting the intrinsic (material) stiffness of the polymeric shell. In contrast, acoustic excitation applies isotropic radial stresses on the entire MB (gas‐shell composite) at MHz frequencies, resulting in an effective stiffness that reflects the coupled oscillatory dynamics of the whole system. Attenuation‐derived parameters are thereby functionally relevant for predicting acoustic behavior, as they describe the collective response of whole MB, e.g., system‐level property. In contrast, AFM, by isolating the intrinsic mechanical characteristics of the shell, is better suited for understanding how formulation parameters control the elastic behavior of MB at the material level. In addition, conventional attenuation‐based models assume smooth, uniform shells represented by effective fitted parameters. However, localized AFM measurements are intrinsically sensitive to shell irregularities, e.g., roughness (Figure 3e), which future models need to incorporate. Therefore, while both approaches report “stiffness”, they probe distinct aspects of MB mechanics and are not directly interchangeable. In this study, we consider AFM‐derived stiffness values as more directly informative for material design, whereas attenuation data reflect the functional acoustic behavior of the intact MB population. The apparent opposing trends thus underscore the complementarity of the two approaches, paving the way for future methodological adjustments.

To investigate the acoustic performance of the different samples in the frequency range relevant to clinical US imaging (3–12 MHz), we characterized the backscattering spectra of individual MB when excited by a transducer with a center frequency of 5 MHz in single pulse mode, applied via a Verasonics Vantage 256 US system. Collected signals were normalized to the number of individually detected MB to obtain the averaged response of a single MB. Both the peak intensities attributed to the fundamental band (3–7 MHz) and the second harmonics band (8–12 MHz) increased with reduced (AFM‐derived) shell stiffness (Figure 5e). Therefore, the second harmonic intensities were calculated to be 11.2, 17.2, and 303.8 µW m^−3^ for E_1_B_2_ MB, B_1_B_2_ MB, and O_1_B_2_ MB, respectively ‒ highlighting up to 27.2‐fold and 17.7‐fold increases in the response of O_1_B_2_ MB compared to E_1_B_2_ MB, and B_1_B_2_ MB, respectively. These results were consistent with the previously obtained NLC sonograms shown in Figure 5b.

In summary, O_1_B_2_ MB with reduced AFM‐derived shell stiffness exhibited 3.1‐fold stronger signal intensities in the NLC mode under US irradiation at the center frequency of 18 MHz and up to 17.7‐fold stronger signal in the second harmonic band under US pulse with the center frequency of 5 MHz compared to conventional B_1_B_2_ MB. In contrast, relatively stiff E_1_B_2_ MB exhibited higher destruction rates at higher power US exposures than traditional B_1_B_2_ MB did. These properties allow us to tailor the US responses as a function of MB composition, and improve their performance for US imaging and US‐mediated drug delivery applications. In parallel, discrepancies between AFM‐ and attenuation‐derived MB stiffness trends highlight the different deformation modes sampled by the two measurements and the need for future efforts to better reconcile these approaches, strengthening their ability to describe both the intrinsic MB shell mechanics and the system‐level (effective) stiffness of intact MB.

### 2.5 Cyanoacrylate Composition Potentiates Microbubble‐Mediated Sonoporation

**Figure 6:**
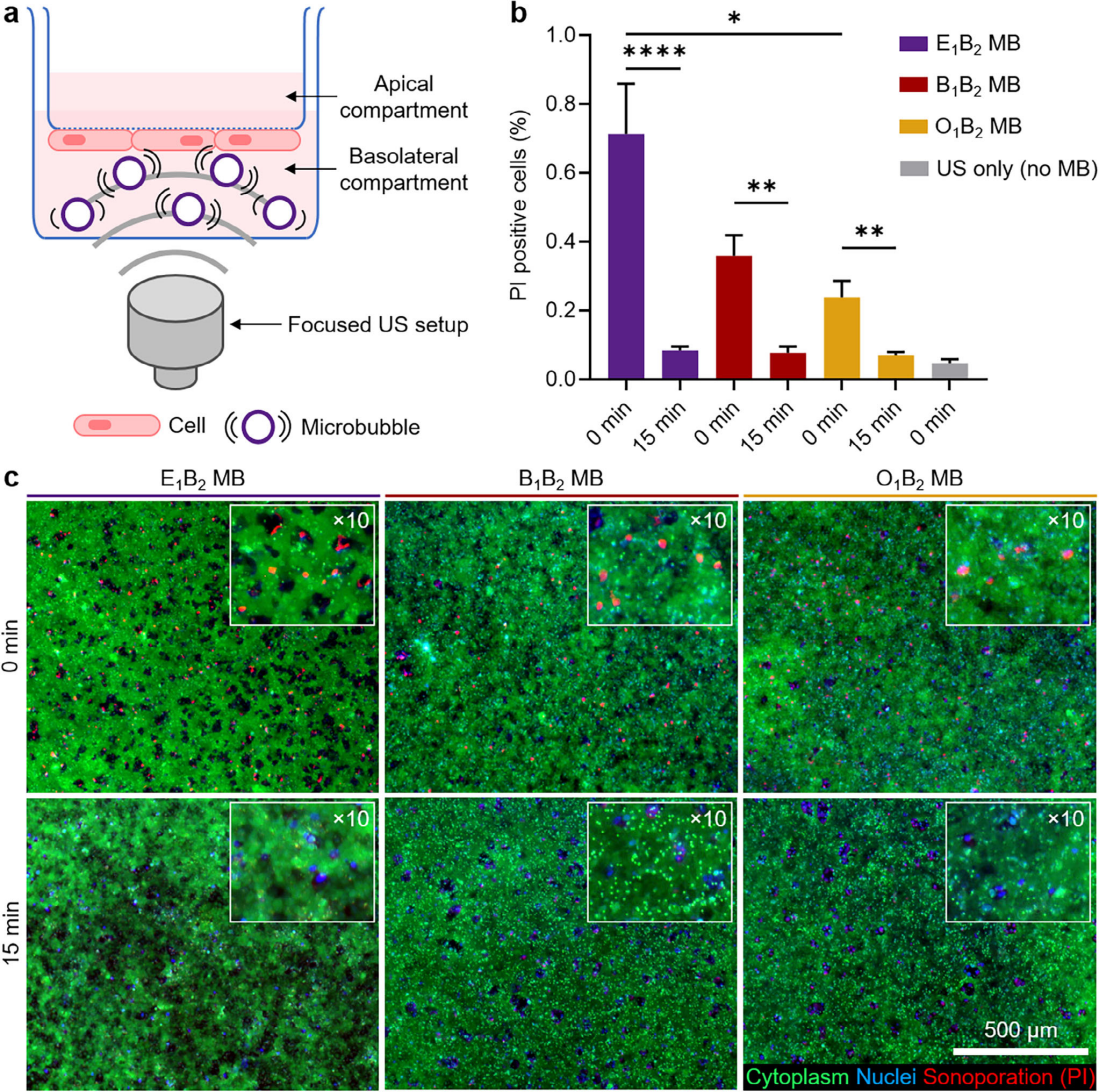
In vitro sonoporation using shell‐optimized PACA MB. a) The experimental setup for the sonoporation experiment comprises a focused US setup equipped with a 1 MHz transducer and peak negative pressure of 650 kPa, as well as a monolayer of Madin‐Darby canine kidney (MDCK) epithelial cells cultured on the basolateral surface of porous cell culture inserts. b) Percentage of propidium iodide (PI) uptake into the monolayer of MDCK cells after PACA MB mediated sonoporation measured at 0 and 15 min after the treatment. c) Corresponding fluorescence micrographs in which nuclei were stained with Hoechst (blue) and live cell cytoplasm was stained with fluorescein diacetate (green), while PI uptake is shown in red, taken 0 and 15 min after the sonoporation with the PACA MB. The inlets show the micrographs with ×10 magnification. (^*^), (^**^) and (^****^) indicate groups that are significantly different with *p* < 0.05, *p* < 0.01 and *p* < 0.0001, respectively (one‐way ANOVA with post hoc Tukey HSD test).

Cell membrane permeability can be facilitated by US‐activated MB near cells with the mechanism called sonoporation, enhancing local drug delivery.^[^ ^2^, ^51^ ^]^ To evaluate this, we tested the sonoporation capabilities of PACA MB using an in vitro experimental model developed at our institute.^[^ ^52^ ^]^ Madin–Darby Canine Kidney (MDCK) epithelial cells were selected as an appropriate model for assessing cell permeability, and were cultured on the basolateral surface of porous cell culture inserts until a confluent epithelial cell monolayer was formed (**Figure** **6**a). The cell layer was exposed to a focused US transducer with a center frequency of 1 MHz in the presence of free‐floating MB, using a previously described custom setup.^[^ ^19^ ^]^ A peak negative pressure of 650 kPa was chosen as a moderate level to facilitate sonoporation using polymeric MB.^[^ ^52^ ^]^

All MB effectively promoted sonoporation in MDCK cells at 1 MHz, as indicated by propidium iodide (PI) cellular uptake, compared to the control group treated with focused US alone without MB (Figure 6b). Among the three samples, E_1_B_2_ MB demonstrated the highest efficacy, resulting in a 3‐fold increase in PI uptake compared to the softer O_1_B_2_ MB, showing superior temporary cell membrane opening. A peak negative pressure of 650 kPa can induce bursting of PBCA MB, as demonstrated in our previous work.^[^ ^52^ ^]^ While shell thickness was comparable across the three developed MB samples, we hypothesize that the higher AFM‐derived stiffness of the E_1_B_2_ MB enhanced its sonoporation performance under the applied US settings. To validate MB bursting, we exposed MB to US using the same setup without inserts containing cell monolayers and measured MB concentrations before and after US pulses. As shown in Figure S13 (Supporting Information), E_1_B_2_ MB exhibited the highest bursting rate (with only a small fraction of MB remaining in 1‒3 µm range), whereas O_1_B_2_ MB demonstrated greater stability, with a larger portion of MB still detectable. While most MB were destroyed across all samples, these results confirm that bursting efficiency varies by composition. Notably, MB bursting events can exert a stronger sonoporation effect on cells than their nondestructive counterparts.^[^ ^52^, ^53^ ^]^

To demonstrate that MB‐induced effect was only temporary, we assessed PI uptake 15 min after applying focused US and MB (Figure 6b). The PI uptake measurements were comparable to the control group treated with focused US alone, showing negligible values. Corresponding fluorescence micrographs shown in Figure 6c for MB‐treated samples highlighted PI uptake immediately after US treatment (0 min) as red dots, whereas only minimal dye uptake was observed when PI exposure was performed 15 min after US treatment, highlighting the transient nature of the sonoporation effect.

In summary, we demonstrated that MB shell composition and AFM‐derived stiffness are key parameters influencing MB‐induced sonoporation. Under peak negative pressure sufficient to cause MB bursting, rigid E_1_B_2_ MB exhibited greater sonoporation capabilities than soft O_1_B_2_ MB. Additionally, for all MB samples, the sonoporation effect was only temporary, which is important for minimizing potential long‐term disruption to cell membrane integrity and reducing the risk of adverse cellular effects. This ensures that the enhanced permeability is controlled and reversible, making the process safe for therapeutic applications.

### 2.6 Shell‐Optimized Microbubbles Provide Superior In Vivo US Imaging Performance

Based on the excellent in vitro US imaging results of O_1_B_2_ MB over B_1_B_2_ MB under preclinical settings (shown in Figure 5), we investigated their performance as US contrast agents in vivo using a preclinical US setup with a central transducer frequency of 18 MHz. For this purpose, eight Balb/cAnNRj mice (four per group) were intravenously injected with 50 µL of MB at a concentration of 2 × 10^9^ MB mL^−1^. Comparable doses of PBCA MB have been safely employed in numerous in vivo studies for both imaging and therapeutic applications.^[^ ^21^, ^22^, ^26^ ^]^ After administration, the circulation and distribution of MB in the liver and kidneys were imaged for 5 min. These organs and time frames were chosen based on previous studies of the pharmacokinetics and biodistribution of PBCA MB, which have blood circulation half‐lives under 5 min, US detection times over 10 min, and show strong contrast signal in the liver and kidneys. Over time, MB are taken up by phagocytes and cleared by the liver and spleen.^[^ ^54^, ^55^ ^]^

**Figure 7:**
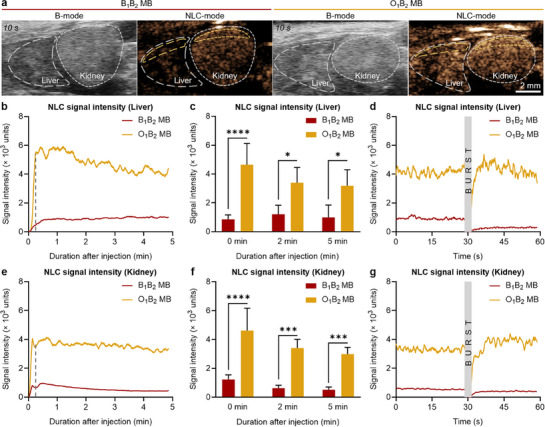
In vivo US imaging performance of shell‐engineered PACA MB. a) US sonograms of mouse liver and kidney in B‐mode and NLC‐mode after i.v. injection. Sonograms were captured 10 s after recording began. Gray dashed lines indicate organ contours, while yellow dashed lines represent regions of interest selected for quantitative analysis. Scale bar: 2 mm. b) NLC signal intensity over time curves of the different MB in the liver. c) Average NLC signal intensity acquired in the liver of mice at different time points after injection. d) NLC signal intensity graph acquired in the liver of mice 5 min after injection and after bursting with 100 % US power. e) NLC signal intensity over time curves of the different MB in the kidneys. f) Average NLC signal intensity acquired in the kidneys of mice at different time points after injection. g) NLC signal intensity graph acquired in the kidneys of the mice 5 min after injection and after bursting with 100 % US power. Measurements were done at 10 % US power. Each group contained four animals. Values represent mean ± standard deviation. (^*^), (^***^) and (^****^) indicate groups that are significantly different with *p* < 0.05, *p* < 0.001 and *p* < 0.0001, respectively (two‐way ANOVA). The dashed gray lines in graphs (b) and (e) indicate the time points corresponding to the acquisition of the representative sonograms displayed in panel (a).

Representative B‐mode and NLC‐mode sonograms of the mouse kidneys and livers post‐injection, as shown in **Figure** **7**a, clearly indicated that the MB synthesized with OCA provided brighter images in NLC‐mode compared to standard B_1_B_2_ MB. This observation is further supported by Figure 7b, which shows a representative graph of the NLC signal intensity over time in the liver, highlighting the enhanced performance of O_1_B_2_ MB over conventional B_1_B_2_ MB. A rapid increase in signal intensity immediately post‐injection was observed for both samples, indicating the presence and accumulation of the MB in the organ. This was followed by a gradual decrease in intensity over the course of 5 min, likely due to MB bursting as they were exposed to US waves. Furthermore, the reduction in signal may also be influenced by shadowing effects.^[^ ^56^ ^]^ Similarly, the mean NLC signal intensities of the samples at different time points after injection (0, 2, and 5 min) demonstrated that the O_1_B_2_ MB exhibited up to 5.4‐fold higher NLC signal compared to B_1_B_2_ MB (Figure 7c). The destruction of MB in vivo was further examined by applying a US destructive pulse with high‐intensity (100 %) power. Immediately after the destructive pulse was applied, a marked drop in signal intensity was recorded in the liver (Figure 7d), confirming the destruction of a substantial portion of MB accumulated within the organ. Within seconds, the signal intensity began to recover, as intact MB from the bloodstream started to replenish the liver vasculature. Notably, the post‐replenishment signal for B_1_B_2_ MB remained lower than pre‐destruction levels, while O_1_B_2_ MB achieved similar intensity levels before and after the pulse. This difference may be attributed to the stronger initial echogenicity of O_1_B_2_ MB, which likely contributed to increased shadowing artifacts prior to bursting. Therefore, the observed recovery in signal intensity may reflect a reduction in shadowing following MB destruction.^[^ ^56^ ^]^

A comparable MB behavior was observed in the kidneys, wherein the signal exhibited a rapid increase following intravenous administration, subsequently undergoing a gradual and sustained reduction (Figure 7e). Notably, the mean NLC signal intensities of the samples in the kidney at different time points after injection (0, 2, and 5 min) demonstrated that the O_1_B_2_ MB showed up to 5.8‐fold higher NLC signal compared to B_1_B_2_ MB (Figure 7f). Following a destructive US pulse, the NLC signal dropped sharply, indicating MB destruction in the renal vasculature (Figure 7g). Rapid signal recovery suggested replenishment by intact MB from the bloodstream, while the comparable pre‐ and post‐pulse intensity levels observed for O_1_B_2_ MB likely resulted from reduced shadowing artifacts following MB destruction.

The collective in vivo findings corroborated the in vitro US imaging results and illustrated that the MB with reduced (AFM‐derived) shell stiffness exhibited superior imaging capabilities in vivo compared to conventional PBCA MB.

**Figure 8:**
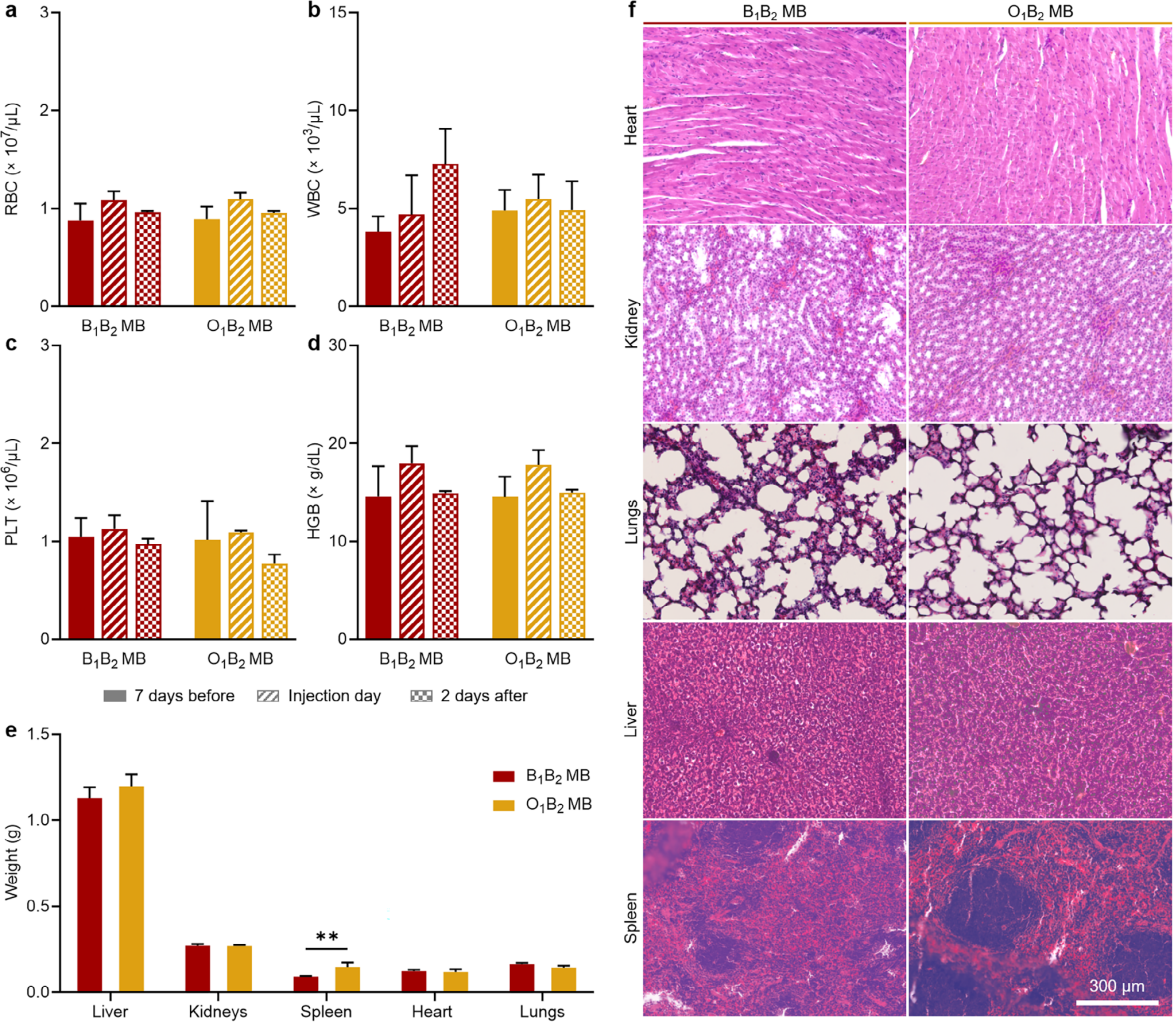
In vivo acute toxicity assesment of the PACA MB. a) Red blood cells (RBC) count, b) white blood cells (WBC) count, c) platelet (PLT) count, and d) hemoglobin (HGB) concentration of blood samples at different time points of the experiment. e) Weight of organs, and f) H&E‐stained micrographs of mouse organs two days after MB administration. Each group contained four animals. Values represent mean ± standard deviation. (^**^) indicates groups that are significantly different with *p* < 0.01 (two‐way ANOVA).

As previously mentioned, BCA‐ and OCA‐based formulations are approved by the American Food and Drug Administration as surgical adhesives, and the safety of PBCA MB has been well established through multiple in vitro and in vivo studies conducted on both small and large animals.^[^ ^38^, ^57^ ^]^ Nevertheless, to confirm the absence of acute toxicity of these newly developed MB, we performed comprehensive blood analysis, physical examination, and histopathological evaluation. Blood samples were collected from the animals 7 days prior to, immediately after, and 2 days following the imaging procedure, and a blood count was performed, which included the quantification of red blood cells (RBC), white blood cells (WBC), platelets (PLT), hemoglobin (HGB), hematocrit (HCT), mean corpuscular hemoglobin (MCH), mean corpuscular hemoglobin concentration (MCHC), and mean corpuscular volume (MCV). Although some slight variations were observed (**Figure** **8**a–d; Figure S14, Supporting Information), all the values were within normal biological ranges.^[^ ^58^, ^59^ ^]^

Two days after MB administration (and in vivo imaging), the animals were euthanized, and relevant organs were harvested for histopathological analysis. After euthanasia, organs were visually examined and showed no signs of distress or damage. Organ weights were measured (Figure 8e), revealing a slight increase in the liver and spleen weights after O_1_B_2_ MB administration. However, all organ weights remained within healthy biological ranges.^[^ ^60^, ^61^ ^]^ This aligns with previous findings that OCA‐based polymer chains degrade more slowly than PBCA due to their increased chain length and hydrophobicity, leading to extended accumulation in organs of the reticuloendothelial system.^[^ ^28^, ^32^ ^]^ Additionally, prior studies have shown similar biodistribution patterns for PACA‐based nanomedicines in tumor‐bearing mice.^[^ ^62^ ^]^

Histopathological analysis was performed by fixing the organs in 4% v/v formalin, followed by dehydration, paraffin embedding, and sectioning. Tissue slices were deparaffinized with xylene and ethanol, then stained with hematoxylin and eosin (H&E) to assess potential acute toxicological responses in host tissues. Figure 8f shows representative H&E micrographs of each sample showing no pathological features. Taken together, these results confirm that both B_1_B_2_ MB and O_1_B_2_ MB samples did not cause acute toxicity or serious adverse effects.

### 2.7 Discussion and Perspectives

Conventional PBCA MB are synthesized via anionic polymerization of BCA, a monomer known for its high reactivity and rapid polymerization.^[^ ^10^ ^]^ Previous reports extensively demonstrated that PBCA MB display a good compromise of properties, namely narrow diameter distribution, high additive loading, bioconjugation capabilities, and adequate acoustic responses, making them reliable contrast agents for functional and molecular US imaging.^[^ ^10^ ^]^ However, these inherent properties are likely limiting factors for more sophisticated therapeutic applications. For instance, the opening of the blood‐brain barrier ideally requires stronger acoustic responses without causing MB destruction,^[^ ^63^ ^]^ which are characteristics displayed by softer MB shells.^[^ ^64^ ^]^

MB acoustic and drug loading capabilities are primarily determined by the shell material and synthesis protocol, and the rapid nature of the BCA polymerization limits our ability to manipulate them through polymer and colloidal chemistry. For example, our preliminary work demonstrated that attempts to manipulate the acoustic properties of MB through colloidal chemistry (e.g., manipulating the surfactant templates during MB synthesis, or partial MB shell hydrolysis) led to significant changes in the MB morphological characteristics, e.g., shell thickness.^[^ ^14^, ^25^ ^]^ Therefore, our new approach presented in this manuscript effectively surmounts these challenges.

This is the first study to precisely engineer the shell of polymeric MB by fabricating the shell material with different monomers that each induce distinct physicochemical properties, including hydrophobicity and rigidity. By strategically combining different components, the resulting MB can display radically different characteristics. For instance, we can manipulate the (AFM‐derived) MB shell stiffness, yielding formulations with high acoustic responses, as demonstrated through US imaging and AFM characterization. These features are highly desired for the blood‐brain barrier opening with therapeutic US setups, which rely on large acoustic oscillation to effectively actuate endothelial cells and allow therapeutics to penetrate across the barrier while minimizing (or even avoiding) potential side effects, such as haemorrhage and inflammatory responses.^[^ ^65^, ^66^, ^67^ ^]^ Conversely, our results demonstrate increased drug loading capacity of newly‐developed formulations while maintaining high drug release rates, which may enhance US‐mediated drug delivery to tumors by facilitating the opening of tumor vasculature through sonoporation and sonopermeation effects.^[^ ^68^ ^]^

Notably, the MB shell engineering was achieved using biocompatible materials already approved by the American Food and Drugs Administration for other applications (e.g., surgical adhesives), while maintaining the desirable features of conventional PBCA MB, namely narrow MB diameter distribution, defined morphology, and shell thickness, and formation of relatively short polymer chains within the MB shells, well below the renal clearance thresholds.^[^ ^10^ ^]^ Thus, this study represents the first attempt to precisely tailor polymeric MB features –, e.g., (AFM‐derived) stiffness – for specific US‐mediated diagnostic and therapeutic applications through shell composition engineering.

## 3 Conclusion 

In summary, we demonstrate that precise manipulation of the polymer chemistry of PACA MB through copolymerization of monomers with different alkyl chain lengths significantly enhances their functional performance. While maintaining the same shell thickness and MB size, the (AFM‐derived) shell stiffness has been tuned by more than 10‐fold, enabling a 2‐fold increase in drug loading capacity, a 3‐fold improvement in sonoporation efficiency, and in vitro 18‐fold and in vivo 6‐fold greater acoustic responses. All‐atom molecular dynamics simulations corroborated these findings by providing mechanistic insights into the enhanced drug loading capabilities. In vivo studies further confirmed the superior acoustic performance and the biocompatibility of the newly developed formulations. These results underscore the critical role of shell engineering in manipulating the polymeric MB features and significantly improving their performance for US imaging, sonoporation, and drug delivery applications.

## 4 Experimental Section

### Materials

Butyl cyanoacrylate (BCA) was purchased from Special Polymer Ltd. (Bulgaria). Ethyl cyanoacrylate (ECA) was purchased from Permabond Engineering Adhesives Ltd. (UK). 2‐octyl cyanoacrylate (OCA), Triton X‐100, gelatin, coumarin 6, dimethyl sulfoxide (DMSO), poly‐*L*‐Lysine (PLL), propidium iodide (PI), fluorescein diacetate (FDA) and Hoechst were purchased from Sigma‐Aldrich (Germany). Dulbecco's modified Eagle's medium (DMEM), phosphate‐buffered saline (PBS), fetal bovine serum (FBS), penicillin‐streptomycin (P/S) antibiotics, ethylenediaminetetraacetic acid (EDTA), trypsin and poly‐*D*‐Lysine were purchased from Thermo Fisher Scientific (Germany). Falcon cell culture inserts (Catalog No. 353096) for the in vitro sonoporation experiment were purchased from Corning (Germany), and US‐compatible Lumox multiwell plates were purchased from Sarstedt AG & Co. KG (Germany). Deionized (DI) water was produced with a PURELAB flex 2 device from ELGA LabWater (Germany) and used for all experiments. All other reagents were of analytical grade.

### Synthesis of Polymeric MB

The synthesis of polymeric MB was based on the previously reported procedure.^[^ ^10^, ^22^ ^]^ Briefly, a 300 mL solution of 1 % (w/v) Triton X‐100 at pH 2.5 was prepared. Next, 44 mM of BCA and 22 mM of corresponding monomer (ECA, BCA, or OCA) were added dropwise to the solution at room temperature. Afterward, the solution was mixed at 10 000 RPM for 1 h with an Ultra‐Turrax T50 (IKA‐Werke, Germany). BCA monomer polymerization during high‐speed stirring of the Triton X‐100 solution resulted in a suspension of air‐filled B_1_B_2_ MB (identical to PBCA MB previously reported by the group^[^ ^10^, ^25^ ^]^), whereas copolymerization of BCA with ECA or OCA resulted in suspensions of air‐filled E_1_B_2_ MB or O_1_B_2_ MB, respectively. To isolate the MB with the desired size (2–3 µm diameter), resulting suspensions were centrifuged three times at 500 RPM for 20 min and the purified MB were resuspended in 80 mL Triton X‐100 0.02 % w/v at pH 7 (storage solution).

### Drug Loading in Polymeric MB

Coumarin 6 was loaded into the polymeric MB as a model drug according to the previously established method.^[^ ^25^, ^26^ ^]^ 5 mg of coumarin 6 was dissolved in 100 µL of DMSO and added to a 10 mL suspension of 10^10^ MB in storage solution. The mixtures were then stirred at 50 RPM overnight. Next, the samples were left undisturbed for at least 6 h to allow the MB to float and form a MB cake. The aqueous solutions underneath the MB cakes were replaced with fresh storage solution multiple times to remove the unloaded drug until no free drug was detected in the solution via fluorescence spectrometry with a TECAN Infinite Pro M200 (Tecan, Switzerland). Finally, the drug‐loaded MB were resuspended in 10 mL of storage solution and stored in the dark for further characterizations. All the procedure was done in the dark to prevent photobleaching and at room temperature.

### Quantification of Concentrations, Size Distributions and Zeta Potentials of Polymeric MB

The concentration and size distribution of the polymeric MB were measured with a Multisizer 4 (Beckmann Coulter, Germany). 5 µL of each MB sample was diluted in 20 mL of ISOTON II isotonic solution (Beckmann Coulter, Germany) and measured in volumetric mode with analytical volume of 50 µL. For each sample, 20 mL of isotonic solution was measured with the same procedure and subtracted from the measurement result as background noise. In addition, size distributions and zeta potential were measured by DLS (dynamic light scattering) using a Zetasizer Nano‐ZS (Malvern Instruments, UK). A scattering angle of 173° and intensity mode were used for DLS measurements. Each sample was diluted with the appropriate storage solution. All measurements were performed at room temperature.

### Characterization of the Polymeric MB Shell Composition

GPC (gel permeation chromatography) and proton NMR (nuclear magnetic resonance spectroscopy) were performed to analyze the shell material. Prior to measurements, MB suspensions were washed three times with DI water to minimize the presence of Triton X‐100 by subsequent steps of MB floatation to the air‐liquid interface and aspiration of the liquid underneath. The MB suspensions were then lyophilized using a benchtop lyophilizer CHRIST Alpha 2‐4 LD plus (Martin Christ Freeze‐Drying Systems GmbH, Germany) to obtain powders of polymer chains that formed the MB shells.

To evaluate the polymer chains (molecular weight and polydispersity index) by GPC, the samples were dissolved in high‐performance liquid chromatographic grade chloroform stabilized with 2‐methyl‐2‐buthene (VWR, USA) after lyophilization. A GPC set up, which included PU‐2080 plus high‐performance pump (Jasco, Germany), RI‐2031 plus refractive index detector (Jasco, Germany), Sedex 85 evaporative light scattering detector (Sedere, France), one pre‐column of 8 × 50 mm^2^ and four SD Plus gel columns of 8 × 300 mm^2^ (MZ Analysentechnik, Germany), were utilized. Measurements were performed at 40 °C and 1 mL min^−1^ flow rate with gel particles of 5 µm and pore width of 50, 100, 1000 and 10000 Å. The calibration was done with polystyrene beads (Polymer Standards Service, Germany).

NMR spectra were recorded on a Bruker Avance III 400 MHz spectrometer (Bruker, Germany) using deuterated chloroform as solvent. Prior to the analysis of MB shell material composition, spectra of BCA monomer and Triton X‐100 surfactant were recorded as a control.

### Quantification of Triton X‐100 in Polymeric MB Shells

The relative amount of Triton X‐100 incorporated into the MB shells was quantified based on the integration of selected proton signals in the NMR spectra, as described below for each formulation.

### E_1_B_2_ MB

The overlapping signal at 4.04 ppm contains contributions from methylene protons of both the ECA and BCA units (assigned as signals 2 and 4, respectively, shown in Figure S3a, Supporting Information) as well as from the CH_2_ group of Triton X‐100 (signal 6 in Figure S2b, Supporting Information for Triton X‐100 alone). The total integral of this combined signal was used to estimate the proportion of Triton X‐100. The Triton X‐100 signal contribution was 0.14, and the total signal intensity (including monomers) was 3.01. The relative Triton content was calculated as:

(1) $$\begin{array}{ccc} \%\;(\mathrm{Triton}\;X-100) & & =\frac{I(\mathrm{Triton}\;X-100)\;}{I(2,4)+I(\mathrm{Triton}\;X-100)}\times \;100\;\%\\ & & =\;\frac{0.14}{3.01}\;\;\times \;100\;\%\;\approx \;5\;\%. \end{array}$$

### B_1_B_2_ MB

Signal *I(2) = 2.16* from BCA units of polymeric chains (shown in Figure S3b, Supporting Information) includes a contribution of 0.20 from the CH_2_ group of Triton X‐100. Therefore, the surfactant content was calculated similarly:

(2) $$\begin{array}{ccc} \%\;(\mathrm{Triton}\;X-100) & = & \frac{I(\mathrm{Triton}\;X-100)}{I(2)+I(\mathrm{Triton}\;X-100)}\times \;100\;\%\\ & = & \frac{0.20}{2.16}\times \;100\;\%\;\approx \;9\;\% \end{array}$$

### O_1_B_2_ MB

The methyl group signal *I(5,13) = 3.00* (signal 5,13 in Figure S3c, Supporting Information) was compared to the aromatic signal of Triton X‐100 at ∼6.75 ppm (signal 5 in Figure S2b, Supporting Information). Since this methyl signal corresponds to three protons, and the Triton aromatic signal corresponds to two protons, the integrals were normalized accordingly:

(3) $$\begin{array}{ccc} \%\;(\mathrm{Triton}\;X-100) & = & \frac{(I(\mathrm{Triton}\;X-100)/2)\;}{\left(I(5,13)/3\right)}\times \;100\;\%\;\\ & & \approx \;\;\frac{0.03}{1.00}\times \;100\;\%=3\;\% \end{array}$$

### Quantification of Co‐Monomer Ratios in Polymeric MB Shells

The molar ratios between BCA and either ECA or OCA were determined using NMR spectra by integrating characteristic proton signals of the respective monomer units. All integrations were normalized such that an integral value of 1.00 corresponds to one proton.

### E_1_B_2_ MB

To determine the ratio of BCA to ECA units (Figure S3a, Supporting Information), signal 7–corresponding to the methyl group (CH_3_) of the BCA unit—was used as an internal reference and manually set to an integral value of 3.00 (i.e., 1.00 per proton). The co‐monomer ratio was calculated using the combined signal at 4.04 ppm, which includes contributions from methylene groups adjacent to the acrylate moieties of both BCA (signal 4) and ECA (signal 2), along with a small contribution from the CH_2_ group of Triton X‐100 (signal 6 in Figure S2b, Supporting Information). After subtracting the Triton contribution (0.14), the corrected integral of the mixed signal was 2.87. Based on the 3.00‐reference scaling, signal 4 accounts for 2.00 protons from BCA, leaving 0.87 protons attributed to ECA. This corresponds to ≈30% ECA and 70% BCA in the polymer backbone.

### O_1_B_2_ MB

Signal 5,13—corresponding to the methyl groups (CH_3_) of both BCA and OCA units—was used as the internal reference and set to an integral value of 3.00. The co‐monomer ratio was determined by comparing signal 2 (CH_2_ group of BCA) with signal 6 (CH group of OCA). Signal 2 also included a Triton X‐100 contribution, which was subtracted (0.08), yielding a corrected integral of 1.40. Since signal 2 represents a CH_2_ group (2 protons), its value was divided by 2 for per‐proton comparison, resulting in 0.70. Signal 6 corresponds to a single proton and had an integral value of 0.25. Using these values, the composition was estimated to be ≈26 % OCA and 74 % BCA in the MB shell.

### Characterization of the Polymeric MB Morphology and Shell Stiffness

CryoSEM (scanning electron cryo‐microscopy) and CLSM (confocal laser scanning microscopy) micrographs were used to measure the shell thickness of polymeric MB, while AFM (atomic force microscopy) was used to evaluate the MB shell stiffness and roughness of polymeric MB.

CryoSEM micrographs of intact polymeric MB were obtained using a FE‐SEM 4800 (Hitachi, Germany) equipped with an Alto 2500 cryo‐transfer system (Gatan GmbH, Germany). Each sample was frozen in liquid nitrogen, sectioned in a cryochamber, and then sublimated for up to 10 min to remove residual water. Micrographs were then taken at 1 kV and 2 µA, and MB characteristics were measured using ImageJ software (National Institute of Health, USA).

For CLSM, polymeric MB loaded with coumarin 6 were mounted with Mowiol (Carl Roth, Germany) on high‐precision glass slides (Marienfeld, Germany) and analyzed using a Leica inverted confocal microscope (TCS SP8 X, Leica Microsystems, Germany) equipped with a plan apochromat 100×/1.40 set at an excitation wavelength of 470 nm and an emission detection range from 491 to 556 nm. MB shell thickness values were measured using Leica Application Suite X 3.7.4. In addition, CLSM micrographs were used to analyze the mean diameter distribution of coumarin 6‐loaded MB samples.

Since AFM measurements in a liquid require a firm attachment of the MB to the substrate, additional preparation steps were implemented. Glass slides were coated with a 1:10 solution (v/v) of PLL in DI water, removing the excess liquid after 15 min, and drying overnight. To attach MB to the substrate, PLL‐coated glass slides were inverted on a 200‐fold diluted MB suspension for 30 min and then rinsed with DI water to remove unattached MB. All AFM measurements were performed in DI water. AFM was performed on a JPK NanoWizard 4XP (Bruker, Germany) mounted on an Axio Observer 5 inverted microscope (Zeiss, Germany). Each sample surface was imaged using a 63× objective and optical overlay to accurately place the cantilever over the MB. Optical images were also taken between force‐distance measurements to define the MB radius and fracture force.

AFM force spectroscopy data were obtained using a CSC12 tipless cantilever (MikroMasch, Bulgaria) with a spring constant of 1 N/m. Reference force curves were recorded on a clean glass slide before and after the MB measurements and were used to determine the sensitivity of the cantilever. A z‐distance of 4 µm was used to ensure no interaction between the MB and the cantilever prior to contact, and a cantilever velocity of 2 µm s^−1^ was maintained during approach and retraction. Force spectroscopy measurements were performed on 30 individual MB from each sample. JPK Data Processing 4.2 software (Brucker, Germany) was used to analyze each force‐distance curve. The stiffness of each MB (*s*) was calculated as a function of force (*F*) divided by deformation (*∆*):

(4) $$s=\frac{F}{\Delta }$$

The individual MB stiffness was derived from the initial linear portion of the curve to avoid instabilities that occur as the force applied to the microbubble increases and can cause shape changes due to MB stretching, buckling, and rupture.^[^ ^69^, ^70^, ^71^ ^]^

The AFM quantitative imaging mode was implemented to image the individual MB surface (and assess MB shell roughness) using a SENSE 70 cantilever (NuNano, UK) with a nominal spring constant of 0.4 N/m. A set point of 0.3 nN was used to avoid MB damage and fracture, and a z‐length of 200 nm and a speed of 40 µm s^−1^ were used to acquire images of individual MB with a size of 3 × 3 µm^2^ and a resolution of 256 × 256 pixels. The images were processed using Mountains SPIP v10.1 software (Digital Surf, France), where a 1 × 1 µm area within each MB was selected and levelled with a second‐order polynomial line fit to account for the overall curvature of the MB, then smoothened with a low‐level median spatial filter. Surface roughness values were derived as root mean square values of the MB surface measured by AFM.

### Quantification of Drug Loading and US‐Mediated Drug Release

To quantify the loading capabilities of polymeric MB samples, each sample was diluted 100‐fold in DMSO, and its fluorescence intensity was measured using a TECAN Infinite Pro M200 (Tecan, Switzerland) set at an excitation wavelength of 467 nm and an emission wavelength of 514 nm.

The average number of drug molecules encapsulated in each MB type was quantified to enable direct comparison across formulations and to maintain consistency with the previous studies,^[^ ^18^, ^25^, ^26^ ^]^ where drug loading was reported on a per‐MB basis.

To measure drug release percentage of polymeric MB, each sample was diluted 500‐fold in storage solution (due to the very low solubility of coumarin 6 in aqueous media) and sonicated for 15 min with an Emmi H60 sonication bath (EMAG AG, Germany). The resulting mixture was then centrifuged at 10 000 RPM for 10 min to remove the MB fragments, and the amount of coumarin 6 dissolved in the supernatant was measured with a TECAN Infinite Pro M200.

The average number of drug molecules encapsulated in each MB type (*N_drug/MB_*), and drug release percentage (*DRP*) were calculated based on the following equations:

(5) $$N_{drug/MB}=\frac{C_{d}}{M_{d}}\times \frac{N_{A}}{C_{MB}}$$

(6) $$DRP=\frac{W_{r}}{W_{d}}\times 100\%$$

where *C_d_* is the concentration of encapsulated drug (mg/mL), *M_d_* is the molecular weight of coumarin 6 (350.43 g mol^−1^), *N_A_* is the Avogadro's number (6.022 × 10^2^ ^3^ mol^−1^), *C_MB_* is the concentration of each sample (MB/mL), *W_r_* is the weight of drug released in solution after sonication, and *W_d_* is the weight of drug loaded in polymeric MB samples.

### All‐Atom Molecular Dynamics Simulations of the Polymeric MB Shell Fragments

All simulations were performed using the Gromacs 2020.6 package.^[^ ^72^ ^]^ The all‐atom optimized potentials for liquid simulations (OPLS‐AA) force field, a commonly utilized approach for simulating diverse polymer systems,^[^ ^73^ ^]^ was employed to describe the interactions between polymeric chains composed of ECA, BCA, and OCA monomeric units, as well as the small molecule coumarin 6 and Triton X‐100 additives. The extended simple point charge (SPC/E) model was employed for the simulation of water molecules.^[^ ^74^ ^]^ The motion equations were solved using the leapfrog integrator, with long‐range electrostatics calculated by the particle mesh Ewald method with a 1.2 nm cutoff.^[^ ^75^ ^]^ Hydrogen bonds were constrained using the linear constraint solver (LINCS) algorithm.^[^ ^76^ ^]^ Simulations were conducted at 27 °C, maintained by a velocity‐rescale thermostat with a time constant of 0.01 ps, and a semi‐isotropic pressure of 1 atm in the vertical direction.^[^ ^77^ ^]^ Pressure was regulated using the Berendsen barostat during the initial equilibrium phase and the Parrinello‐Rahman barostat for subsequent equilibrium, with a time constant of 0.5 ps.^[^ ^78^, ^79^ ^]^ In order to ensure the accuracy and reliability of the results, periodic boundary conditions were applied in all directions. The simulation process entailed an initial brief run with a 0.001 fs timestep for 10^6^ steps, followed by a more extended equilibration phase utilizing a 2 fs timestep. The total simulation time was 500 ns for the equilibration phase (representing the polymeric MB shell fragments) and 1000 ns for the coumarin 6 loading phase (representing the diffusion of coumarin 6 into polymeric MB shell fragments), with the final 50 ns allocated to the calculation of chain equilibrium properties.

### In Vitro US Imaging and Acoustic Characterization of the Polymeric MB

The MB acoustic properties were examined with a Vevo 3100 preclinical US device (VisualSonics, Canada) after embedding the MB in gelatin phantoms. Briefly, for each sample, a mixture of 2 % (w/v) of gelatin and 1.1 × 10^5^ MB mL^−1^ was prepared, as this concentration was selected to avoid acoustic shadowing based on the previous experiments.^[^ ^14^, ^26^ ^]^ The mixture was then embedded in 10 % (w/v) gelatin molds and left in 4 °C overnight. Subsequently, the phantoms were examined with the MX250 transducer operating at a center frequency of 18 MHz in non‐linear contrast (NLC) mode, which is based on pulse amplitude modulation. Initially, the signal intensity of each specimen was quantified at 4 % power (mechanical index of 0.03). The peak negative pressure at this setting is estimated to be in the range of 100  kPa, with spatial peak temporal average intensity remaining below 29 mW cm^−2^.^[^ ^80^, ^81^ ^]^ These settings were chosen to assess MB acoustic responses while minimizing destruction. Subsequently, a second sonogram was recorded at the same 4 % power after each sample had been exposed to higher‐power US (10 %, 15 %, 25 %, and 100 %, corresponding to mechanical indices of 0.07, 0.105, 0.175, and 0.7, respectively) for 5 s. MB destruction rates were calculated as follows:

(7) $$\mathrm{MB}\;\mathrm{destruction}\;\mathrm{rate}\left(\%\right)=\frac{A-B}{A-C}\times 100\;\%$$

where *A* is the mean signal intensity of the sample before higher acoustic power (recorded at 4 % power), *B* is the mean signal intensity of the sample after higher acoustic power (recorded at 4 % power), *C* is the mean signal intensity of the gelatin phantom (background signal, recorded at 4 % power). The mean signal intensities were measured in a region of interest of 25 mm^2^ at the probe focus area (set at 10 mm depth) with the Vevo LAB software.

The same US setup was used for the flow phantom experiments, similar to.^[^ ^9^ ^]^ A custom‐made plastic box (88 mm length × 58 mm width × 90 mm depth) was designed to provide sufficient space for transducer placement on the top surface. Two 2 mm diameter holes were positioned 15 mm below the top plane on opposing shorter walls. A 2 mm stainless steel tube was inserted through these holes to form a channel, and a 10 % (w/v) gelatin solution was poured and allowed to solidify overnight. Prior to the experiment, the tube was removed to create a flow channel, and a peristaltic pump (Gilson Inc., France) was connected via plastic tubing. A suspension of 5 × 10^5^ MB in 15 mL of storage solution was circulated through the system for 5 min in a closed loop at a constant flow rate of ≈2.2 mL min^−1^, sonograms were recorded with a framerate of 20 frames per second. The acoustic power was set to 4 %, corresponding to a mechanical index of 0.03. Clinical‐grade contrast agents SonoVue (Bracco S.p.A., Italy) and Optison (GE Healthcare, USA) were used as controls. SonoVue was reconstituted according to the manufacturer's instructions, while Optison was gently agitated until no visible residue remained at the bottom of the vial. Signal intensities were measured in a region of interest of 7.2 mm^2^ at the probe focus area (set at 10 mm depth) with the Vevo LAB software. Curve smoothing was performed using a second‐order polynomial and a window of 60 neighboring points on each side.

Attenuation measurements were performed using a custom experimental setup as previously reported.^[^ ^47^, ^48^ ^]^ Briefly, each MB sample was diluted to several orders of 10^6^ particles in a volume of ≈17 cm^3^ and placed in a glass chamber (2 × 2 × 5 cm) under continuous stirring. The glass chamber had two opposite openings where the transmitting and receiving flat wide‐band V311 transducers (Olympus, Japan) were in contact with the liquid. The distance between the two transducers was fixed and equal to 2 cm. US signals were generated by a waveform generator with frequencies ranging from 0.5 to 20 MHz as successive sinusoidal bursts with 4 µs steps of 250 kHz burst frequency. The negative pressure was maintained at a level of 14 kPa or lower to permit the MB to undergo low‐amplitude and linear oscillations. The US signal and the attenuation spectra were controlled and acquired by home‐made software based on LabView (National Instruments Corporation, USA).

The analysis of the attenuation measurements is based on the theory from Hoff et al.^[^ ^49^ ^]^ The total attenuation of the MB cloud is the sum of the individual extinction cross sections of single MB σ(*R*,  ω) which depends on the radius *R* and angular driving frequency ω = 2π*f*. The particle size distribution *n*(*R*) is used to numerically integrate the attenuation:

(8) $$\alpha \left(\omega \right)=\int_{0}^{\infty }n\left(R\right)\sigma \left(R,\omega \right)dR$$

Considering the low‐pressure amplitude used in the attenuation measurements, a linear model can be used to describe the volumetric oscillations of a single MB. A correction for the thermal damping of the gas core is applied.^[^ ^50^ ^]^ The resulting resonance angular frequency is:

(9) $$\omega_{0}=\frac{1}{R}\sqrt{\frac{3\kappa p_{l}}{\rho_{l}}+\frac{4\chi }{\rho_{l}R_{0}}}$$

where *p_g_* is the hydrostatic pressure, ρ_*l*_ is the liquid density, κ is the polytropic exponent, and χ = 3 *G_s_d_s_* is the reduced shell stiffness (*G_s_* is the shear modulus of the material and *d_s_* is the shell thickness). The linear system is damped by a factor δ, with contributions of fluid viscosity, shell viscosity, thermal effects and acoustic radiation. the work of Hoff et al. was referred for full derivations.^[^ ^49^ ^]^ The shell viscosity has the most significant effect:

(10) $$\delta_{s}=\frac{4\frac{\kappa_{s}}{R}}{\omega_{0}\rho_{l}R^{2}}$$

where and κ_*s*_ = 3 µ_*s*_ *d_s_* is the reduced shell viscosity (µ_*s*_ is the shear viscosity of the material). The linear oscillations model leads to an expression for the extinction cross‐section:

(11) $$\sigma \left(R,\omega \right)=4\pi R^{2}\frac{c\delta }{R\omega_{0}}\frac{\Omega^{2}}{{\left(1-\Omega^{2}\right)}^{2}+\Omega^{2}\delta^{2}}$$

where *c* is the sound velocity and Ω = ω/ω_0_. Using the measured particle size distributions, the model parameters χ and κ_*s*_ are optimized to fit the normalized attenuation curves. A half‐bandwidth evaluation of the summed squared residuals yielded the approximate confidence intervals.

Backscattering spectra of PACA MB (power density profiles in B‐mode) were evaluated with a Verasonics Vantage 256 US device (Verasonics, Inc, USA) equipped with the linear array L12‐3v transducer transmitting one cycle pulse with a center frequency of 5 MHz. Briefly, a box containing 400 mL of degassed water with a sound absorbing mat at the bottom was prepared. MB were diluted and added and stirred constantly so that spatially separated responses from individual MB could be analyzed. For each sample, 100 independent images were acquired in B‐mode with a focused sequence at a focal depth of 18 mm, a mechanical index of 0.208, and a maximum rarefraction pressure of 635 kPa. 313 individual E_1_B_2_ MB, 2240 individual B_1_B_2_ MB, and 2622 individual O_1_B_2_ MB were localized in a window of 12.24 mm with the focal depth in the center and the RF signal at the MB position was cropped. For each cropped sample signal, the periodogram was calculated from the acquired radio frequency data. The power density spectra were plotted by averaging the values from all the MB.

### Modeling of the Polymeric MB Destruction Rate Under US Pulses

The MB destruction rate (*D_MB_*) under US pulses was calculated on the basis of void nucleation‐damage theory, as previously described in ref. [46]. In this approach, the MB system (1.1 × 10^5^ MB mL^−1^, as taken from the experiment conditions) was theoretically modeled a representative polymer chain, with the number of Kuhn segments equal to the total number of Kuhn segments of all MB in the system.

(12)

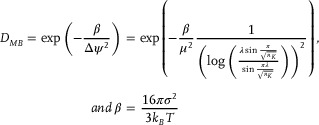

where Δψ is the change in free energy of the polymer of the MB, *k_B_* is the Boltzmann constant, *T* is the absolute temperature, and σ is the surface tension of the polymer used to make the MB, *n*_κ_ is the number of Kuhn segments in the representative polymer chain determined by fitting based on GPC results of polymeric chains that compose MB shells and shell thickness analysis, and λ is the radial stretch on the MB system (Figure S11, Supporting Information).

The quantity in the denominator is the square of the change in free energy of the representative polymer chain under deformation,^[^ ^82^ ^]^ and the shear modulus µ of the MB system can be obtained from the experimental results of the shell stiffness of a single MB obtained by AFM. The argument of the exponential function is the energy barrier for damage growth of the MB system, it can be observed that when the stretch in the MB system reaches the limit of chain extensibility of its representative polymer chain, the change in free energy of the polymer is infinite, so that the MB destruction ratio is 100 %.

The radial stretch on the MB system can be approximated as $\lambda \;\approx \lambda_{0}\;\sqrt{P_{ratio}}$, where λ_0_ is the stretch on the MB system at the lowest acoustic power (4 %), and *P_ratio_* represents the acoustic power ratio relative to the lowest value applied to the MB system (namely 10, 15, 25, and 100 % to 4 %).

Based on experimental data of MB polymer chain composition, shell thickness and stiffness values, the following estimated parameters were used for modeling: $\frac{\beta }{\mu^{2}}$ = 1.96 × 10^−2^, *n*_κ_ = 21.2, and λ_0_ = 1.2649 for E_1_B_2_ MB, $\frac{\beta }{\mu^{2}}$ = 2.716 × 10^−6^, *n*_κ_ = 1594.1, and λ_0_ = 1.0924 for B_1_B_2_ MB, $\frac{\beta }{\mu^{2}}$ = 4.793 × 10^−7^, *n*_κ_ = 4297.5, and λ_0_ = 1.0449 for O_1_B_2_ MB, respectively.

### In Vitro Sonoporation of Cell Monolayers

The in vitro sonoporation experiment was performed according to the previously established protocol in the group described in.^[^ ^52^ ^]^ Briefly, Madin‐Darby canine kidney (MDCK) epithelial cells (passages 13–25) were cultured in DMEM with 10% FBS and 1% P/S antibiotics until 80–90 % confluence. Cells were detached using 0.05 % EDTA (5 min) and 0.25% trypsin (4 min), then resuspended at 2 × 10^6^ cells mL^−1^ in 80 µL of culture medium. The suspension was added to the basolateral surface of poly‐D‐lysine‐coated (0.1 mg mL^−1^) cell culture inserts by inverting the inserts in the plate. Inserts were incubated upside down for 4 h to facilitate cell attachment. Afterward, the plate was reverted, and fresh medium (1 mL) was added to the basolateral compartment, with 0.5 mL to the apical compartment. Cells were cultured on the inserts for two nights to reach ≈90 % confluence, with one medium change during this period. Each insert with cells is referred to as a sample.

A custom‐built focused US setup equipped with a V314‐SU‐F1.00in‐PTF transducer with 1 MHz center frequency and 25 mm focal length (Evident Europe GmbH, Germany) was used to irradiate MB along with the samples for 2 s with a peak negative pressure of 650 kPa, a pulse repetition frequency of 2 kHz, and 10 cycles per pulse. Peak negative pressures were in situ calibrated with a 1‐mm needle hydrophone placed inside the Lumox well plate, as characterized in.^[^ ^19^ ^]^ The transducer was fixed in an upright vertical position in a water tank filled with DI water. Then, a sample was placed in a well plate, and the entire plate was mounted on top of the transducer with the bottom of the plate in the water. The distance between the transducer surface and the sample was adjusted to 25 mm so that the focal point was aligned with the sample.

Once the sample was placed in the well and aligned with the transducer, it was exposed to focused US as described. Sonoporation events were detected by adding 300 µL of staining solution per sample to a US‐compatible well plate. The solution contained PI (red, 2 mg/mL), FDA (green, 5 mg mL^−1^), and Hoechst (blue, 1 µg/mL) in DMEM. PI detects sonoporation by entering cells with perforated membranes and fluorescing red upon intercalation with RNA/DNA, while FDA verifies cell viability, and Hoechst counterstains nuclei as a reference. For “0 min” measurements, the staining solution and MB (2 × 10^7^ MB in 50 µL DMEM) were added to the sample prior to US exposure, the sample was exposed to focused US, left in the staining solution for 2 min after exposure, and then washed twice with PBS. No MB was applied to the control sample (“US only”). For “15 min” measurements, DMEM and MB were added prior to US exposure, and the sample was exposed to US. After exposure, the sample was incubated for 15 min, DMEM was replaced with the staining solution, and the sample was left for 2 min before being washed twice with PBS.

The insert membranes were cut out, placed on microscopy slides, and immediately imaged using the Axio Imager M2 fluorescence microscope (Carl Zeiss, Germany) at 10× magnification. Images were analyzed with ImageJ (National Institutes of Health, USA). Each color channel was processed separately. To quantify PI‐positive cells, Otsu's thresholding method was applied to highlight regions with PI fluorescence, followed by particle counting. The number of PI positive cells $N_{PI}^{cells}$ was normalized to the total cell count in the imaged region, calculated as the sum of PI positive $N_{PI}^{cells}$ and FDA positive cells $N_{FDA}^{cells}$ as shown below:

(13) $$PI\;positive\;cells=\frac{N_{PI}^{cells}}{N_{PI}^{cells}+N_{FDA}^{cells}}*100\%$$

Additionally, equal amounts of MB (2 × 10^7^ MB) were introduced into wells without inserts containing cell monolayers, and Coulter counter measurements were performed before and after ultrasound exposure to assess MB bursting. These experiments were conducted in triplicate, and representative averaged diameter distributions were plotted.

### In Vivo US Imaging and Biocompatibility

In vivo experiments were conducted using eight female Balb/cAnNRj mice (Janvier Labs, France), aged 10–12 weeks (4 per group), to assess polymeric MB performance and safety under biological conditions. The study was approved by the German State Office for Nature, Environment, and Consumer Protection (LANUV, approval number: AZ 81‐02.04.2020.A204) and complied with institutional guidelines, EU Directive 2010/63/EU, and German federal animal protection laws. Humane care followed the “Guide for the Care and Use of Laboratory Animals.”. A power analysis using G^*^Power software (two‐tailed unpaired t‐test) determined that 4 animals per group were needed, giving a statistical significance of 0.80 (power 1‐β_err_ prob).

Mice were housed in groups of four with spruce granulate bedding (Lignocel, JRS, Germany) under specific pathogen‐free conditions. Environmental controls included a 12‐h light/dark cycle, 20–24 °C temperature, and 45 – 65% humidity. Animals had ad libitum access to standard pellets (Sniff GmbH, Germany) and water. Individual mice were marked for identification and assigned to groups by assigning random numbers in Excel. Daily health monitoring and experiments were conducted by two experienced unblinded researchers. No exclusion criteria were applied, and all the gathered data were reported in the manuscript.

Mice were anesthetized with inhalation anesthesia (induction: 5 % isoflurane in 95 % oxygen; maintenance: 2 % isoflurane in 98 % oxygen) for immobilization during injection and imaging. A 50 µL dose of MB (2 × 10^9^ MB mL^−1^) was administered via a tail vein catheter. Mice were positioned on their left side, and the right flank – spanning from below the lungs to the intestines—was shaved to minimize imaging artifacts. The transducer was placed transversely (perpendicular to the spine), directing the beam cranially and dorsally through the skin, right kidney, and liver. This orientation enabled simultaneous visualization of both organs within a single imaging plane beneath the skin. US gel was used to ensure good acoustic contact without excessive pressure. Circulation and distribution of MB in the liver and kidneys were imaged using a Vevo 3100 preclinical US device (VisualSonics, Canada) with an MX250 transducer (18 MHz, 10 % power) for 5 min with a framerate of 10 frames per second. A 10 % acoustic power setting was used to balance image resolution while minimizing extensive MB destruction, consistent with previous reports.^[^ ^17^, ^21^, ^26^ ^]^ After that, a 60 s cineloop was recorded under the same settings, with a 1 s burst (100 % US power) at the 30 s mark. Body temperature was maintained using a temperature‐controlled platform.

Signal intensities were quantified using Vevo LAB software. To account for differences in NLC signal intensity between MB samples, regions of interest were chosen to be of comparable size (≈2 mm^3^) across all samples. In addition, regions of interest were selected near the US probe, covering areas adjacent to the upper kidneys and liver, to minimize potential shadowing artifacts and signals from the skin. Motion during imaging sessions was primarily attributable to normal respiration, which caused minor periodic displacements of the region of interest; no abrupt or large bulk motions were observed. While no motion correction was applied, curve smoothing was performed using a second‐order polynomial and a window of 30 neighboring points on each side, similar to the previous reports.^[^ ^21^, ^26^ ^]^ For each animal, average signal intensities were extracted, and both intra‐animal variability (standard deviation of signal intensity within each sample) and inter‐animal variability (variation of average values across animals) were considered in the analysis.

To assess the toxicological profile of the polymeric MB, blood samples were collected 7 days before, immediately after, and 2 days after the US imaging procedure for analysis. At the end of the study, mice were euthanized under deep isoflurane anesthesia via cervical dislocation. The heart, liver, spleen, lungs, and kidneys were harvested for histopathological analysis.

### Histopathological Analysis

Collected organs were fixed in 4 % formalin overnight, dehydrated using a Leica TP 1020 tissue processor (Leica, Germany), and embedded in paraffin. The paraffin blocks were then sectioned into 8 µm thick slices with an HM 430 sliding microtome (Thermo Fisher Scientific, USA) and dried at 37 °C overnight. Sections were heated at 60 °C for 2 h, then deparaffinized with xylene and serial ethanol solutions.

Hematoxylin and Eosin (H&E) staining was performed to visualize nuclei and extracellular matrix. Deparaffinized sections were incubated with Hematoxylin solution (Carl Roth GmbH, Germany) for 15 min, washed with water for 15 min, stained with acidified Eosin solution (Carl Roth GmbH, Germany) for 30 s, and rinsed again for 5 min. Samples were dehydrated with ethanol and xylene, then mounted with a Vitro‐clud adhesive (R. Langenbrinck GmbH, Germany). Micrographs were acquired at 20× magnification using a Vectra 3.0 automated microscope (PerkinElmer, USA).

### Statistical Analysis

For each sample, three different batches were synthesized, and each measurement was performed at least three times. Results are reported as mean ± standard deviation. ANOVA analysis with post hoc Tukey HSD test or two‐way ANOVA analysis were performed with the GraphPad Prism 8. *p*‐values lower than 0.05 were considered as statistically significant; (ns), (^*^), (^**^), (^***^) and (^****^) indicate groups with *p* > 0.05, *p* < 0.05, *p* < 0.01, *p* < 0.001, and *p* < 0.0001, respectively.

## Conflict Of Interest

F. Kiessling, T. Lammers and A. Rix are among the co‐founders of the SonoMAC GmbH that produces polymeric microbubbles. F. Kiessling is a consultant of Fujifilm Visualsonics. The other authors have no relevant conflicts of interest to declare.

## Author Contributions

R.A.B., T.L. and R.M.P. conceived the project and designed the research; R.A.B. carried out the synthesis and characterization of the PACA MB, collected and interpreted the data, and wrote the first manuscript draft. M.M. and E.R. assisted in the experiments; J.Bl. established the model of in vitro sonoporation and conducted the experiment; A.R. established the protocols for in vivo US imaging experiments; V.S.P. and R.A.Gu. conducted all‐atom molecular dynamics simulations; J.K., and M.P. performed the polymeric composition characterization experiments; C.B., S.R., and L.C. performed the microscopy for MB morphology and (AFM‐derived) stiffness characterization; V.N.K. conducted modeling of the polymeric MB destruction rate under US pulses; F.D., Th.L., and E.S. contributed to in vitro acoustic characterization of polymeric MB; A.R., R.Z., J.Ba., and S.K. contributed to in vivo experiment performance and analysis; V.K., M.I., G.P., G.S., T.V., L.De‐L., R.Gö., A.H., I.P., F.K., T.L. and R.M.P. provided scientific guidance. All authors read and revised the manuscript.

## Supporting information



Supporting Information

## Data Availability

The data that support the findings of this study are available from the corresponding author upon reasonable request.

## References

[1] F. Kiessling , S. Fokong , P. Koczera , W. Lederle , T. Lammers , J. Nucl. Med. 2012, 53, 345. 22393225 10.2967/jnumed.111.099754

[2] K. Kooiman , H. J. Vos , M. Versluis , N. de Jong , Adv. Drug Delivery Rev. 2014, 72, 28. 10.1016/j.addr.2014.03.003 24667643

[3] J. Yan , B. Huang , J. Tonko , M. Toulemonde , J. Hansen‐Shearer , Q. Tan , K. Riemer , K. Ntagiantas , R. A. Chowdhury , P. D. Lambiase , R. Senior , M.‐X. Tang , Nat. Biomed. Eng. 2024, 8, 689. 38710839 10.1038/s41551-024-01206-6 PMC11250254

[4] S. Fleig , Z. A. Magnuska , P. Koczera , J. Salewski , S. Djudjaj , G. Schmitz , F. Kiessling , npj Imaging 2024, 2, 22. 40604097 10.1038/s44303-024-00023-5 PMC12118757

[5] J. K. Willmann , L. Bonomo , A. C. Testa , P. Rinaldi , G. Rindi , K. S. Valluru , G. Petrone , M. Martini , A. M. Lutz , S. S. Gambhir , J. Clin. Oncol. 2017, 35, 2133. 28291391 10.1200/JCO.2016.70.8594 PMC5493049

[6] M. Smeenge , F. Tranquart , C. K. Mannaerts , T. M. de Reijke , M. J. van de Vijver , M. P. Laguna , S. Pochon , J. J. M. C. H. de la Rosette , H. Wijkstra , Invest. Radiol. 2017, 52, 419. 28257340 10.1097/RLI.0000000000000362

[7] E. Stride , C. Coussios , Nat. Rev. Phys. 2019, 1, 495.

[8] A. Upadhyay , S. V. Dalvi , Ultrasound Med. Biol. 2019, 45, 301. 30527395 10.1016/j.ultrasmedbio.2018.09.022

[9] T. M. Estifeeva , A. M. Nechaeva , I. M. Le‐Deygen , A. M. Adelyanov , I. V. Grigoryan , V. S. Petrovskii , I. I. Potemkin , A. A. Abramov , A. V. Prosvirnin , E. A. Sencha , D. A. Borozdenko , R. A. Barmin , Y. O. Mezhuev , D. A. Gorin , P. G. Rudakovskaya , Biomater. Adv. 2025, 166, 214074. 39447238 10.1016/j.bioadv.2024.214074

[10] P. Koczera , L. Appold , Y. Shi , M. Liu , A. Dasgupta , V. Pathak , T. Ojha , S. Fokong , Z. Wu , M. van Zandvoort , O. Iranzo , A. J. C. Kuehne , A. Pich , F. Kiessling , T. Lammers , J. Controlled Release 2017, 259, 128. 10.1016/j.jconrel.2017.03.006 PMC5528138 28279799

[11] R. A. Barmin , M. J. Moosavifar , A. Dasgupta , A. Herrmann , F. Kiessling , R. M. Pallares , T. Lammers , Chem. Sci. 2023, 14, 11941. 37969594 10.1039/d3sc04339h PMC10631124

[12] A. Bauer , M. Blomley , E. Leen , D. Cosgrove , R. Schlief , Eur. Radiol. 1999, 9, S349. 10602927 10.1007/pl00014072

[13] K. Wei , L. Crouse , J. Weiss , F. Villanueva , N. B. Schiller , T. Z. Naqvi , R. Siegel , M. Monaghan , J. Goldman , P. Aggarwal , H. Feigenbaum , A. DeMaria , Am. J. Cardiol. 2003, 91, 1293. 12767419 10.1016/s0002-9149(03)00316-3

[14] R. A. Barmin , A. Dasgupta , A. Rix , M. Weiler , L. Appold , S. Rütten , F. Padilla , A. J. C. Kuehne , A. Pich , L. De Laporte , F. Kiessling , R. M. Pallares , T. Lammers , ACS Biomater. Sci. Eng. 2024, 10, 75. 36315422 10.1021/acsbiomaterials.2c01021

[15] T. Ojha , V. Pathak , N. Drude , M. Weiler , D. Rommel , S. Rütten , B. Geinitz , M. J. van Steenbergen , G. Storm , F. Kiessling , T. Lammers , Pharmaceutics 2019, 11. 10.3390/pharmaceutics11090433 PMC6781551 31454967

[16] M. Palmowski , J. Huppert , G. Ladewig , P. Hauff , M. Reinhardt , M. M. Mueller , E. C. Woenne , J. W. Jenne , M. Maurer , G. W. Kauffmann , W. Semmler , F. Kiessling , Mol. Cancer Ther. 2008, 7, 101. 18202013 10.1158/1535-7163.MCT-07-0409

[17] J. Chen , B. Wang , A. Dasgupta , C. Porte , L. Eckardt , J. Qi , M. Weiler , T. Lammers , A. Rix , Y. Shi , F. Kiessling , J. Nanobiotechnology 2024, 22, 528. 39218888 10.1186/s12951-024-02806-9 PMC11367926

[18] R. A. Barmin , M. Moosavifar , R. Zhang , S. Rütten , S. Thoröe‐Boveleth , E. Rama , T. Ojha , F. Kiessling , T. Lammers , R. M. Pallares , J. Mater. Chem. B 2024, 12, 2511. 38334758 10.1039/d3tb02950f PMC10916536

[19] J. Chen , B. Wang , Y. Wang , H. Radermacher , J. Qi , J. Momoh , T. Lammers , Y. Shi , A. Rix , F. Kiessling , Adv. Sci. 2024, 11, 2306139. 10.1002/advs.202306139 PMC11022722 38342634

[20] J. N. May , S. K. Golombek , M. Baues , A. Dasgupta , N. Drude , A. Rix , D. Rommel , S. Von Stillfried , L. Appold , R. Pola , M. Pechar , L. Van Bloois , G. Storm , A. J. C. Kuehne , F. Gremse , B. Theek , F. Kiessling , T. Lammers , Theranostics 2020, 10, 1948. 32042346 10.7150/thno.41161 PMC6993230

[21] A. Dasgupta , T. Sun , R. Palomba , E. Rama , Y. Zhang , C. Power , D. Moeckel , M. Liu , A. Sarode , M. Weiler , A. Motta , C. Porte , Z. Magnuska , A. Said Elshafei , R. Barmin , A. Graham , A. McClelland , D. Rommel , E. Stickeler , F. Kiessling , R. M. Pallares , L. De Laporte , P. Decuzzi , N. McDannold , S. Mitragotri , T. Lammers , Proc. Natl. Acad. Sci 2023, 120, 2218847120. 10.1073/pnas.2218847120 PMC10068850 36940339

[22] A. Dasgupta , T. Sun , E. Rama , A. Motta , Y. Zhang , C. Power , D. Moeckel , S.‐M. Fletcher , M. Moosavifar , R. Barmin , C. Porte , E. M. Buhl , C. Bastard , R. M. Pallares , F. Kiessling , N. McDannold , S. Mitragotri , T. Lammers , Adv. Mater. 2023, 35, 2308150. 10.1002/adma.202308150 PMC11238272 37949438

[23] M. Xuan , J. Fan , V. N. Khiêm , M. Zou , K.‐O. Brenske , A. Mourran , R. Vinokur , L. Zheng , M. Itskov , R. Göstl , A. Herrmann , Adv. Mater. 2023, 35, 2305130. 10.1002/adma.202305130 37494284

[24] J. Hahmann , A. Ishaqat , T. Lammers , A. Herrmann , Angew. Chem., Int. Ed. 2024, 63, 202317112. 10.1002/anie.202317112 38197549

[25] R. A. Barmin , A. Dasgupta , C. Bastard , L. De Laporte , S. Rütten , M. Weiler , F. Kiessling , T. Lammers , R. M. Pallares , Mol. Pharmaceutics 2022, 19, 3256. 10.1021/acs.molpharmaceut.2c00416 35905480

[26] M. Moosavifar , R. A. Barmin , E. Rama , A. Rix , R. A. Gumerov , T. Lisson , C. Bastard , S. Rütten , N. Avraham‐Radermacher , J. Koehler , M. Pohl , V. Kulkarni , J. Baier , S. Koletnik , R. Zhang , A. Dasgupta , A. Motta , M. Weiler , I. I. Potemkin , G. Schmitz , F. Kiessling , T. Lammers , R. M. Pallares , Adv. Sci. 2024, 11, 2404385. 10.1002/advs.202404385 PMC11516050 39207095

[27] R. A. Barmin , J. Köhler , M. Pohl , B. Becker , F. Kiessling , T. Lammers , A. T. Poortinga , R. M. Pallares , Chem. Commun. 2024. 10.1039/d4cc03572k PMC11427993 39328011

[28] Y. Barkan , M. Levinman , I. Veprinsky‐Zuzuliya , T. Tsach , E. Merqioul , G. Blum , A. J. Domb , A. Basu , Acta Biomater. 2017, 48, 390. 27826005 10.1016/j.actbio.2016.11.011

[29] P. Valadbaigi , R. Ettelaie , A. N. Kulak , B. S. Murray , J. Colloid Interface Sci. 2019, 536, 618. 30391904 10.1016/j.jcis.2018.10.004

[30] C. Duffy , P. B. Zetterlund , F. Aldabbagh , Molecules 2018, 23. 10.3390/molecules23020465 PMC6017548 29461508

[31] M. A. Rajora , A. Dhaliwal , M. Zheng , V. Choi , M. Overchuk , J. W. H. Lou , C. Pellow , D. Goertz , J. Chen , G. Zheng , Adv. Sci. 2024, 11, 2304453. 10.1002/advs.202304453 PMC10811482 38032129

[32] C.‐Y. Huang , Y.‐D. Lee , Int. J. Pharm. 2006, 325, 132. 16857330 10.1016/j.ijpharm.2006.06.008

[33] Z. Denchev , M. Tomanova , A. Lederer , J. Polym. Sci. Part A Polym. Chem. 2008, 46, 5142.

[34] B.‐L. Keller , C. A. Lohmann , S. O. Kyeremateng , G. Fricker , Polymers (Basel) 2022, 14, 998. 35267821 10.3390/polym14050998 PMC8912508

[35] E. Glynos , V. Koutsos , W. N. McDicken , C. M. Moran , S. D. Pye , J. A. Ross , V. Sboros , Langmuir 2009, 25, 7514. 19379000 10.1021/la900317d

[36] C. C. Chen , S.‐Y. Wu , J. D. Finan , B. Morrison , E. E. Konofagou , IEEE Trans. Ultrason. Ferroelectr. Freq. Control 2013, 60, 524. 23475918 10.1109/TUFFC.2013.2594 PMC4123865

[37] R. Nadal , B. P. Valderrama , J. Bellmunt , Nat. Rev. Clin. Oncol. 2024, 21, 8. 37945764 10.1038/s41571-023-00826-2

[38] S. Fokong , B. Theek , Z. Wu , P. Koczera , L. Appold , S. Jorge , U. Resch‐Genger , M. van Zandvoort , G. Storm , F. Kiessling , T. Lammers , J. Controlled Release 2012, 163, 75. 10.1016/j.jconrel.2012.05.007 22580225

[39] E. E. Meyer , K. J. Rosenberg , J. Israelachvili , Proc. Natl. Acad. Sci. USA 2006, 103, 15739. 17023540 10.1073/pnas.0606422103 PMC1635073

[40] S. A. Hollingsworth , R. O. Dror , Neuron 2018, 99, 1129. 30236283 10.1016/j.neuron.2018.08.011 PMC6209097

[41] A. P. Singh , H. Tanaka , Y. Miyazaki , S. Nagano , W. Shinoda , J. Chem. Theory Comput. 2025, 21, 6226. 40493020 10.1021/acs.jctc.5c00498

[42] S. Kotopoulis , M. Popa , M. Mayoral Safont , E. Murvold , R. Haugse , A. Langer , G. Dimcevski , C. Lam , T. Bjånes , O. H. Gilja , E. M. Cormack , Pharmaceutics 2022, 14, 98. 35056994 10.3390/pharmaceutics14010098 PMC8777813

[43] P. J. Martinez , J. J. Song , J. I. Castillo , J. DeSisto , K.‐H. Song , A. L. Green , M. Borden , ACS Biomater. Sci. Eng. 2024, 10, 7451. 39497639 10.1021/acsbiomaterials.4c00777 PMC12399041

[44] S. Koppolu , P. V. Chitnis , J. Mamou , J. S. Allen , J. A. Ketterling , IEEE Trans. Ultrason. Ferroelectr. Freq. Control 2015, 62, 494. 25935932 10.1109/tuffc.2014.006828 PMC4998738

[45] A. J. Sojahrood , H. Haghi , Q. Li , T. M. Porter , R. Karshafian , M. C. Kolios , Ultrason. Sonochem. 2020, 66, 105070. 32279052 10.1016/j.ultsonch.2020.105070

[46] V. N. Khiêm , M. Itskov , Int. J. Plast. 2018, 102, 1, 10.1016/j.ijplas.2017.11.001.

[47] F. Domenici , F. Brasili , L. Oddo , B. Cerroni , A. Bedini , F. Bordi , G. Paradossi , J. Colloid Interface Sci. 2019, 540, 185. 30640066 10.1016/j.jcis.2018.12.110

[48] V. Da Ros , L. Oddo , Y. Toumia , E. Guida , S. Minosse , L. Strigari , S. Strolin , G. Paolani , F. Di Giuliano , R. Floris , F. Garaci , S. Dolci , G. Paradossi , F. Domenici , Pharmaceutics 2023, 15. 10.3390/pharmaceutics15010217 PMC9862136 36678846

[49] L. Hoff , P. C. Sontum , J. M. Hovem , J. Acoust. Soc. Am. 2000, 107, 2272. 10790053 10.1121/1.428557

[50] A. Prosperetti , L. A. Crum , K. W. Commander , J. Acoust. Soc. Am. 1988, 83, 502.

[51] S. Snipstad , K. Vikedal , M. Maardalen , A. Kurbatskaya , E. Sulheim , C. de L. Davies , Adv. Drug Delivery Rev. 2021, 177, 113847. 10.1016/j.addr.2021.113847 34182018

[52] J. Blöck , H. Li , G. Collado‐Lara , K. Kooiman , A. Rix , J. Chen , C. Hark , H. Radermacher , C. Porte , F. Kiessling , ACS Appl. Bio Mater. 2025, 8, 1240. 10.1021/acsabm.4c01551 PMC11836932 39900350

[53] S. Liu , Y. Zhang , Y. Liu , W. Wang , S. Gao , W. Yuan , Z. Sun , L. Liu , C. Wang , Br. J. Cancer 2023, 128, 715. 36463323 10.1038/s41416-022-02076-y PMC9977958

[54] M. Palmowski , B. Morgenstern , P. Hauff , M. Reinhardt , J. Huppert , M. Maurer , E. C. Woenne , S. Doerk , G. Ladewig , J. W. Jenne , S. Delorme , L. Grenacher , P. Hallscheidt , G. W. Kauffmann , W. Semmler , F. Kiessling , Invest. Radiol. 2008, 43, 162. 18301312 10.1097/RLI.0b013e31815a251b

[55] K. T. Warzecha , M. Bartneck , D. Möckel , L. Appold , C. Ergen , W. Al Rawashdeh , F. Gremse , P. M. Niemietz , W. Jahnen‐Dechent , C. Trautwein , F. Kiessling , T. Lammers , F. Tacke , Adv. Biosyst. 2018, 2, 1800002. 29876517 10.1002/adbi.201800002 PMC5985946

[56] J. Chen , Z. Zhu , Ultrasound Med. Biol. 2006, 32, 961. 16785017 10.1016/j.ultrasmedbio.2006.01.018

[57] A. Rix , S. Fokong , S. Heringer , R. Pjontek , L. Kabelitz , B. Theek , M.‐A. Brockmann , M. Wiesmann , F. Kiessling , Invest. Radiol. 2016, 51, 767. 27119438 10.1097/RLI.0000000000000282

[58] G. Silva‐Santana , J. C. Bax , D. C. S. Fernandes , D. T. L. Bacellar , C. Hooper , A. A. S. O. Dias , C. B. Silva , A. M. de Souza , S. Ramos , R. A. Santos , T. R. Pinto , M. A. Ramão , A. L. Mattos‐Guaraldi , Anim. Model. Exp. Med. 2020, 3, 304. 10.1002/ame2.12139 PMC7824965 33532705

[59] C. J. Layssol‐Lamour , J.‐E. Sarry , J.‐P. D. Braun , C. Trumel , N. H. Bourgès‐Abella , J. Am. Assoc. Lab. Anim. Sci. 2021, 60, 4. 33046180 10.30802/AALAS-JAALAS-20-000020 PMC7831346

[60] J. J. Jiménez‐Alonso , E. Guillén‐Mancina , J. M. Calderón‐Montaño , V. Jiménez‐González , P. Díaz‐Ortega , E. Burgos‐Morón , M. López‐Lázaro , Nutrients 2022, 14. 10.3390/nu14163378 PMC9412877 36014884

[61] J. M. Calderón‐Montaño , E. Guillén‐Mancina , J. J. Jiménez‐Alonso , V. Jiménez‐González , E. Burgos‐Morón , A. Mate , M. C. Pérez‐Guerrero , M. López‐Lázaro , Int. J. Mol. Sci. 2022, 23. 10.3390/ijms232416132 PMC9783696 36555771

[62] A. D. Pandya , T.‐G. Iversen , S. Moestue , M. T. Grinde , Ý. Mørch , S. Snipstad , A. K. O. Åslund , G. F. Øy , W. Kildal , O. Engebråten , K. Sandvig , T. Skotland , G. M. Mælandsmo , Nanomaterials 2021, 11. 10.3390/nano11051140 PMC8145722 33924869

[63] Y. Guo , H. Lee , C. Kim , C. Park , A. Yamamichi , P. Chuntova , M. Gallus , M. O. Bernabeu , H. Okada , H. Jo , C. Arvanitis , Nat. Commun. 2024, 15, 8021. 39271721 10.1038/s41467-024-52329-y PMC11399249

[64] A. Dasgupta , M. Liu , T. Ojha , G. Storm , F. Kiessling , T. Lammers , Drug Discov. Today Technol. 2016, 20, 41. 27986222 10.1016/j.ddtec.2016.07.007 PMC5166975

[65] D. McMahon , K. Hynynen , Theranostics 2017, 7, 3989. 29109793 10.7150/thno.21630 PMC5667420

[66] N. Todd , C. Angolano , C. Ferran , A. Devor , D. Borsook , N. McDannold , J. Controlled Release 2020, 324, 450. 10.1016/j.jconrel.2020.05.040 PMC7429281 32470359

[67] R. Ji , M. E. Karakatsani , M. Burgess , M. Smith , M. F. Murillo , E. E. Konofagou , J. Controlled Release 2021, 337, 458. 10.1016/j.jconrel.2021.07.042 PMC8440441 34324895

[68] S. Snipstad , E. Sulheim , C. de Lange Davies , C. Moonen , G. Storm , F. Kiessling , R. Schmid , T. Lammers , Expert Opin. Drug Deliv. 2018, 15, 1249. 30415585 10.1080/17425247.2018.1547279

[69] F. Dubreuil , N. Elsner , A. Fery , Eur. Phys. J. E 2003, 12, 215. 15007658 10.1140/epje/i2003-10056-0

[70] O. I. Vinogradova , J. Phys. Condens. Matter 2004, 16, R1105.

[71] E. Glynos , V. Sboros , V. Koutsos , Mater. Sci. Eng. B 2009, 165, 231.

[72] M. J. Abraham , T. Murtola , R. Schulz , S. Páll , J. C. Smith , B. Hess , E. Lindahl , SoftwareX 2015, 1‐2, 19.

[73] G. A. Kaminski , R. A. Friesner , J. Tirado‐Rives , W. L. Jorgensen , J. Phys. Chem. B 2001, 105, 6474.

[74] H. J. C. Berendsen , J. R. Grigera , T. P. Straatsma , J. Phys. Chem. 1987, 91, 6269.

[75] U. Essmann , L. Perera , M. L. Berkowitz , T. Darden , H. Lee , L. G. Pedersen , J. Chem. Phys. 1995, 103, 8577.

[76] B. Hess , J. Chem. Theory Comput. 2008, 4, 116. 26619985 10.1021/ct700200b

[77] G. Bussi , D. Donadio , M. Parrinello , J. Chem. Phys. 2007, 126, 14101. 10.1063/1.2408420 17212484

[78] H. J. C. Berendsen , J. P. M. Postma , W. F. van Gunsteren , A. DiNola , J. R. Haak , J. Chem. Phys. 1984, 81, 3684.

[79] M. Parrinello , A. Rahman , J. Appl. Phys. 1981, 52, 7182.

[80] P. S. Sheeran , Y. Daghighi , K. Yoo , R. Williams , E. Cherin , F. S. Foster , P. N. Burns , Ultrasound Med. Biol. 2016, 42, 795. 26725168 10.1016/j.ultrasmedbio.2015.11.012

[81] D. Wegierak , M. B. Cooley , R. Perera , W. J. Wulftange , U. A. Gurkan , M. C. Kolios , A. A. Exner , IEEE Trans. Med. Imaging 2024, 43, 2370. 38329864 10.1109/TMI.2024.3364076 PMC11234354

[82] V. N. Khiêm , M. Itskov , J. Mech. Phys. Solids 2016, 95, 254.

