# Supplementary material for: Microbubble Shell Stiffness Engineering Enhances Ultrasound Imaging, Drug Delivery, and Sonoporation
Source: Adv Mater. 2025 Nov 17;38(6):e07655. doi: 10.1002/adma.202507655 (PMC12848657; doi:10.1002/adma.202507655)
Supplement: Supplementary file 1 — Supporting Information [file ADMA-38-e07655-s001.docx]

**– SUPPORTING INFORMATION –**

**Microbubble Shell Stiffness Engineering Enhances Ultrasound Imaging, Drug Delivery and Sonoporation**

Roman A. Barmin,^1^ MirJavad Moosavifar,^1^ Elena Rama,^1^ Julia Blöck,^1^ Anne Rix,^1^ Vladislav S. Petrovskii,^2^
Rustam A. Gumerov,^3^ Jens Köhler,^3^ Michael Pohl,^3^ Céline Bastard,^3,4,5^ Stephan Rütten,^6^ Laura Charlton,^7^
Vu Ngoc Khiêm,^8^ Fabio Domenici,^9^ Thomas Lisson,^10^ Ekaterina Savina,^1^ Rui Zhang,^1^ Jasmin Baier,^1^
Susanne Koletnik,^1^ Vasileios Koutsos,^7^ Mikhail Itskov,^8^ Gaio Paradossi,^9^ Georg Schmitz,^10^ Tina Vermonden,^11^
Laura De Laporte,^3,4,5^ Robert Göstl,^3,4,12^ Andreas Herrmann,^3,4^ Igor I. Potemkin,^3^ Fabian Kiessling,^1^
Twan Lammers,^1,*^ and Roger M. Pallares^1,*^

^1^ Institute for Experimental Molecular Imaging, RWTH Aachen University Hospital, Aachen 52074, Germany

^2^ Institute for Bioengineering of Catalonia, Barcelona 08028, Spain

^3^ DWI – Leibniz Institute for Interactive Materials, Aachen 52074, Germany

^4^ Institute of Technical and Macromolecular Chemistry, RWTH Aachen University, Aachen 52074, Germany

^5^ Department of Advanced Materials for Biomedicine, Institute of Applied Medical Engineering, RWTH Aachen University, Aachen 52074, Germany

^6^ Electron Microscope Facility, RWTH Aachen University Hospital, Aachen 52074, Germany

^7^ School of Engineering, Institute for Materials and Processes, University of Edinburgh, Edinburgh EH9 3FB, UK

^8^ Department of Continuum Mechanics, RWTH Aachen University, Aachen 52062, Germany

^9^ Department of Chemical Science and Technologies, University of Rome “Tor Vergata”, Rome 00133, Italy

^10^ Chair for Medical Engineering, Faculty of Electrical Engineering and Information Technology, Ruhr University Bochum, Bochum 44801, Germany

^11^ Division of Pharmaceutics, Utrecht Institute for Pharmaceutical Sciences (UIPS), Utrecht University, Utrecht 3508 TB, the Netherlands

^12^ Department of Chemistry and Biology, University of Wuppertal, Wuppertal 42119, Germany

*Corresponding authors: [rmoltopallar@ukaachen.de](mailto:rmoltopallar@ukaachen.de); [tlammers@ukaachen.de](mailto:tlammers@ukaachen.de)

Table of Contents

[Figure S1. Physicochemical properties of MB S3](#_Toc204954211)

[Figure S2. NMR spectra of the BCA monomer and Triton X-100 S4](#_Toc204954212)

[Figure S3. NMR spectra of the polymeric MB. Spectra of (a) E_1_B_2_ MB, (b) B_1_B_2_ MB, and (c) O_1_B_2_ MB with peak assignments. S5](#_Toc204954213)

[Figure S4. Representative wide-area cryoSEM and CLSM micrographs of the MB samples S6](#_Toc204954214)

[Figure S5. Individual data points of MB shell thickness and stiffness measurements S7](#_Toc204954215)

[Figure S6. Representative optical micrographs of individual MB from each sample under progressively increasing force applied S8](#_Toc204954216)

[Table S1. Drug molecules per individual MB and drug loading per 1 × 10^9^ MB S9](#_Toc204954217)

[Table S2. The contents of the simulation boxes for all samples S10](#_Toc204954218)

[Figure S7. Simulation snapshots of the polymeric MB shells and corresponding density profiles S11](#_Toc204954219)

[Figure S8. Quantified mean signal intensities of the polymeric MB at 4 % in B-mode S12](#_Toc204954220)

[Figure S9. Acoustic stability assessment of MB in the flow phantom S13](#_Toc204954221)

[Figure S10. Experimental and simulated destruction rates of the PACA MB S14](#_Toc204954222)

[Figure S11. Graphical explanation of MB destruction rate simulations S15](#_Toc204954223)

[Figure S12. Acoustic attenuation spectra of the MB at different concentrations S16](#_Toc204954224)

[Figure S13. Representative MB diameter distributions before and after US setup exposure S17](#_Toc204954225)

[Figure S14. Mouse blood analysis after MB administration at specified timepoints S18](#_Toc204954226)

| 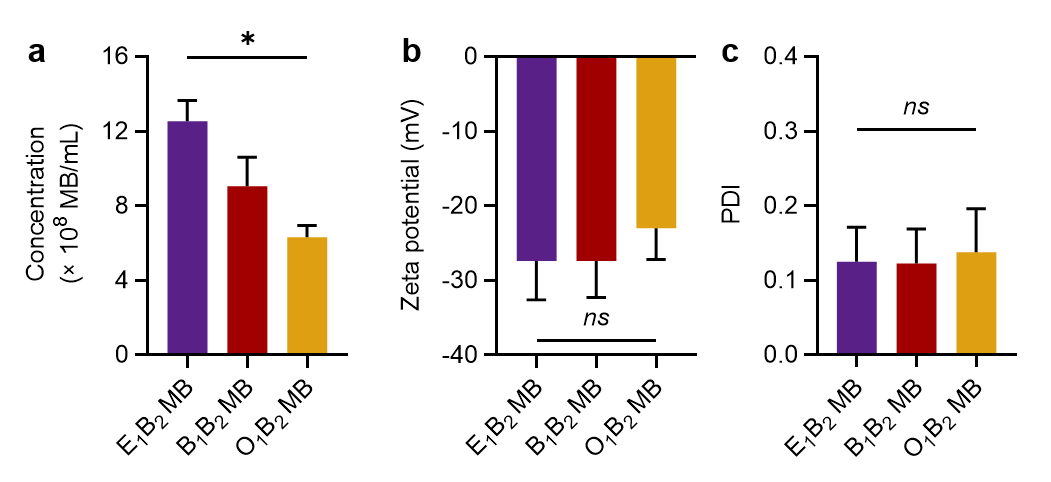 **Figure S1.** **Physicochemical properties of MB.** (**a**) Concentration, (**b**) zeta potential, and (**c**) polydispersity indices (PDI) extracted from DLS measurements. Data represent averages from three independently synthesized batches, with each measurement performed at least three times. (*) indicates groups that are significantly different with p < 0.05; (ns) indicates groups that are not significantly different with p > 0.05 (one-way ANOVA with post hoc Tukey HSD test). |
| --- |

| **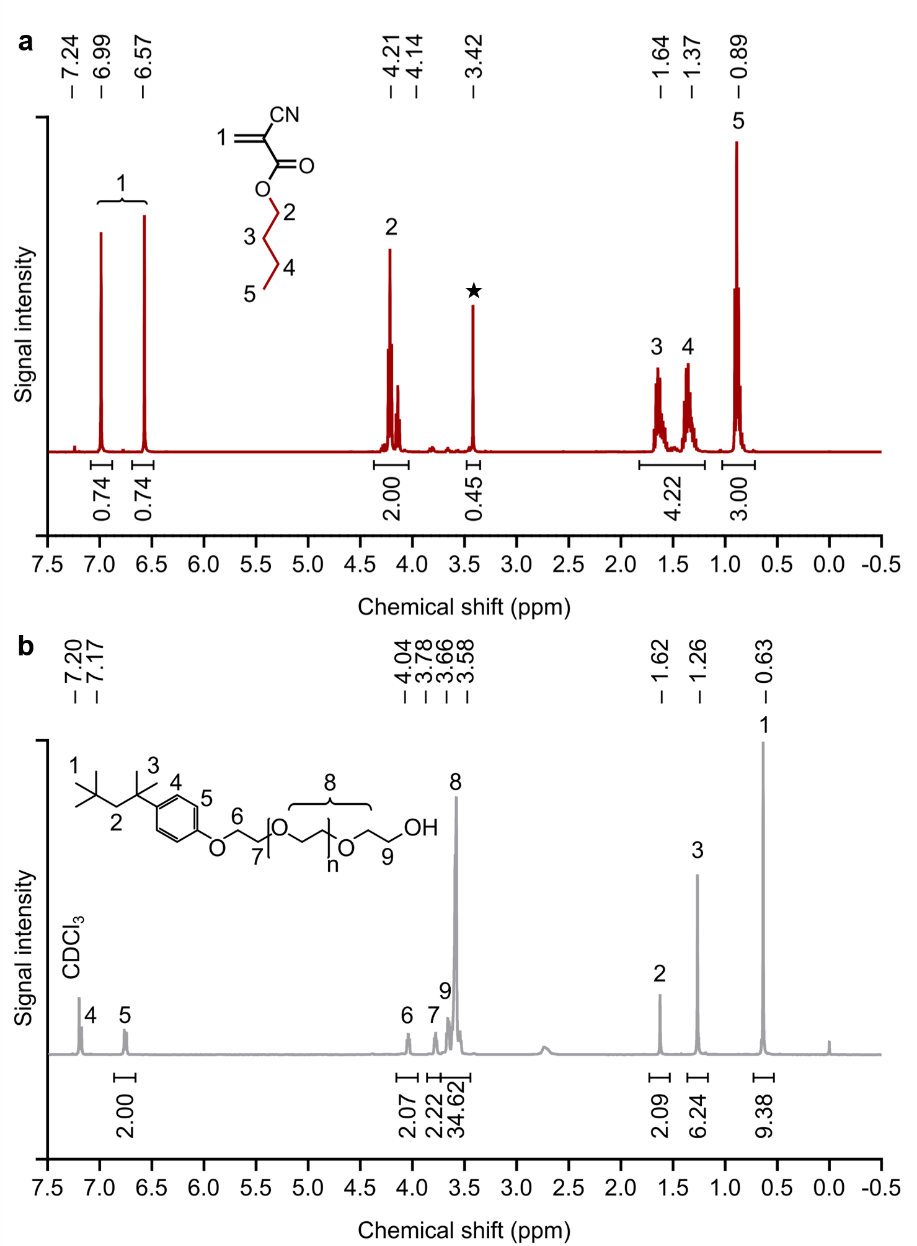**  **Figure S2.** **NMR spectra of the BCA monomer and Triton X-100.** Spectra of (**a**) BCA monomer and (**b**) Triton X-100 with peak assignments. The star indicates the peak that is due to the presence of impurities in the BCA monomer source. |
| --- |

| 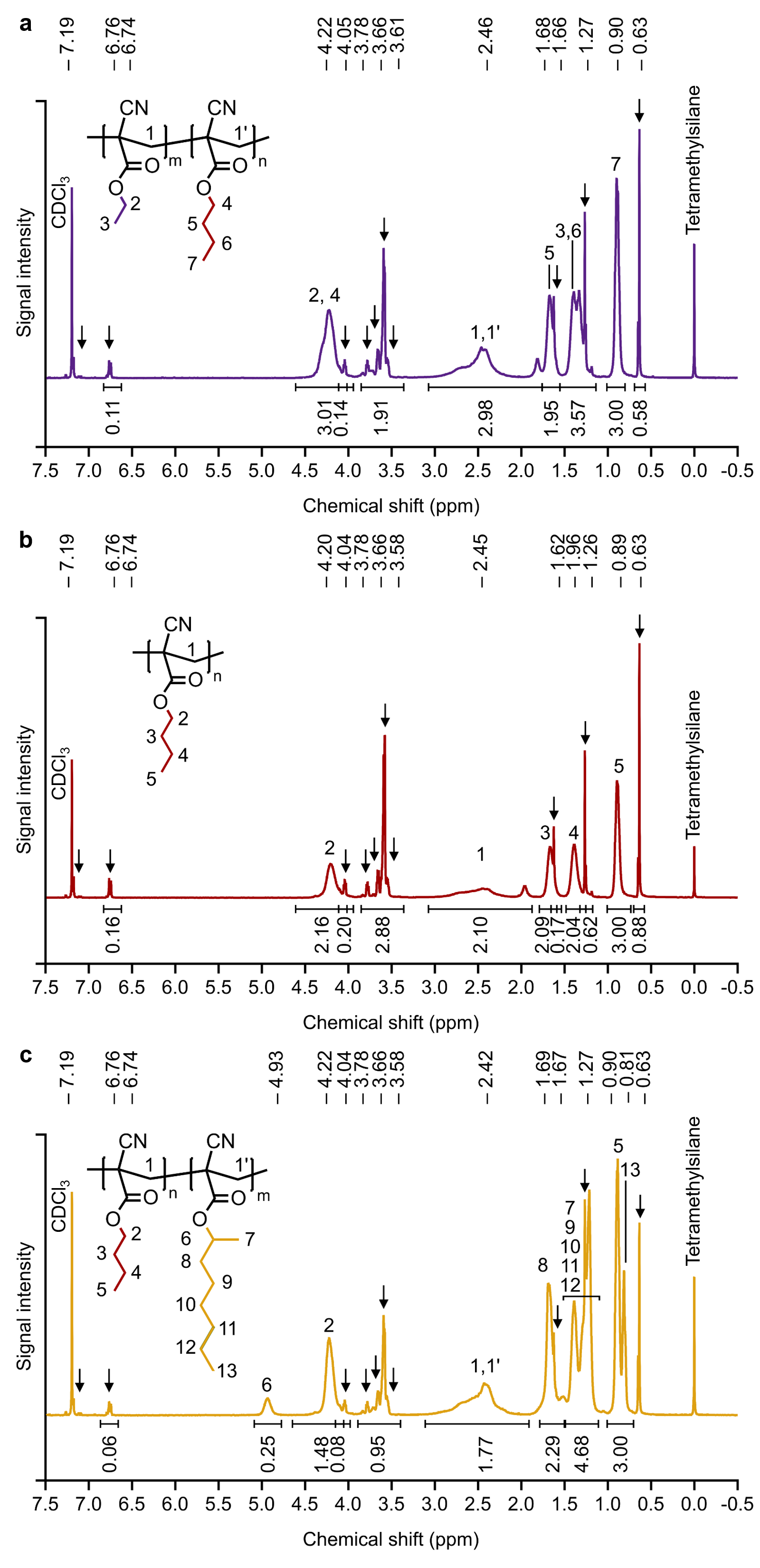**Figure S3. NMR spectra of the polymeric MB.** Spectra of (**a**) E_1_B_2_ MB, (b) B_1_B_2_ MB, and (c) O_1_B_2_ MB with peak assignments. The arrows indicate the peaks that are due to the presence of Triton X-100 in the samples. |
| --- |

| 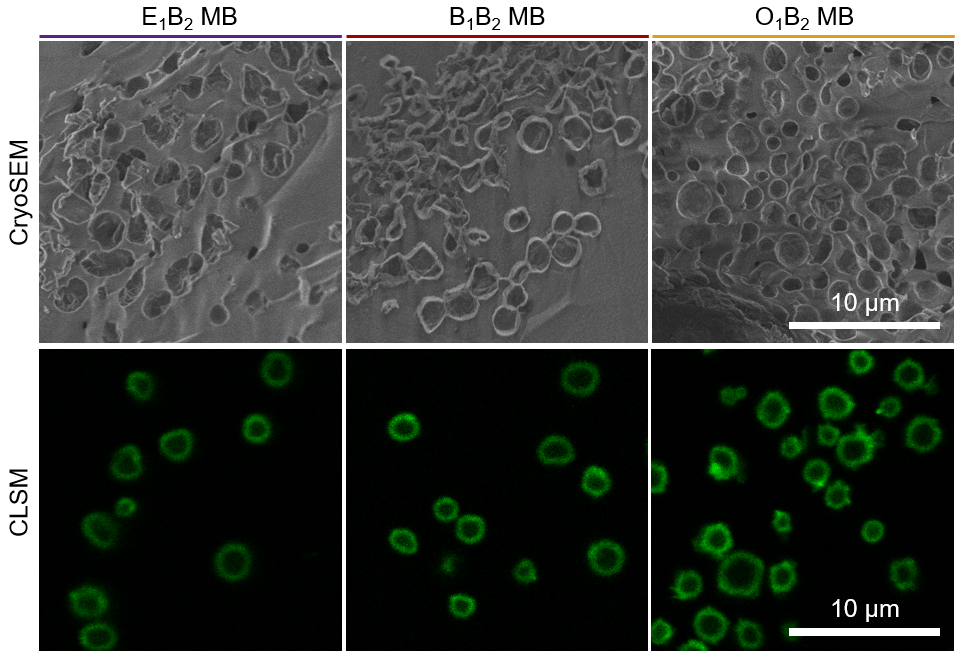  **Figure S4.** **Representative wide-area cryoSEM and CLSM micrographs of the MB samples.** CLSM images are displayed as the signal intensity levels observed during the measurements. |
| --- |

| 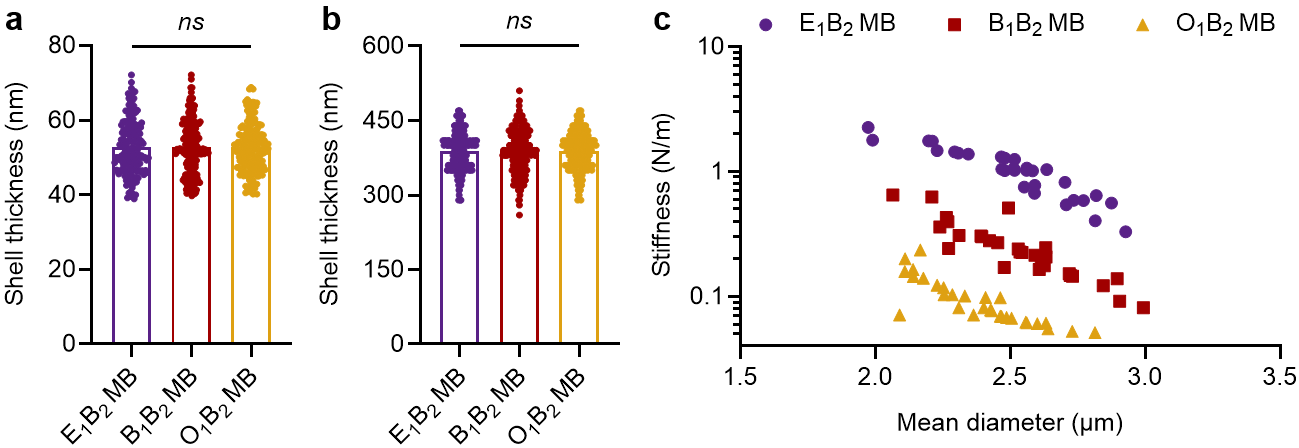 **Figure S5. Individual data points of MB shell thickness and stiffness measurements.** Shell thickness data points obtained from (**a**) cryoSEM images (n = 180 MB or higher per sample), (**b**) CLSM images (n = 270 MB or higher per sample), and (**c**) stiffness values corresponding to the mean diameter of each measured MB shown on logarithmic scale (n = 30 per sample). (*ns*) indicates groups that are not significantly different with p > 0.05 (one-way ANOVA with post hoc Tukey HSD test). |
| --- |

| 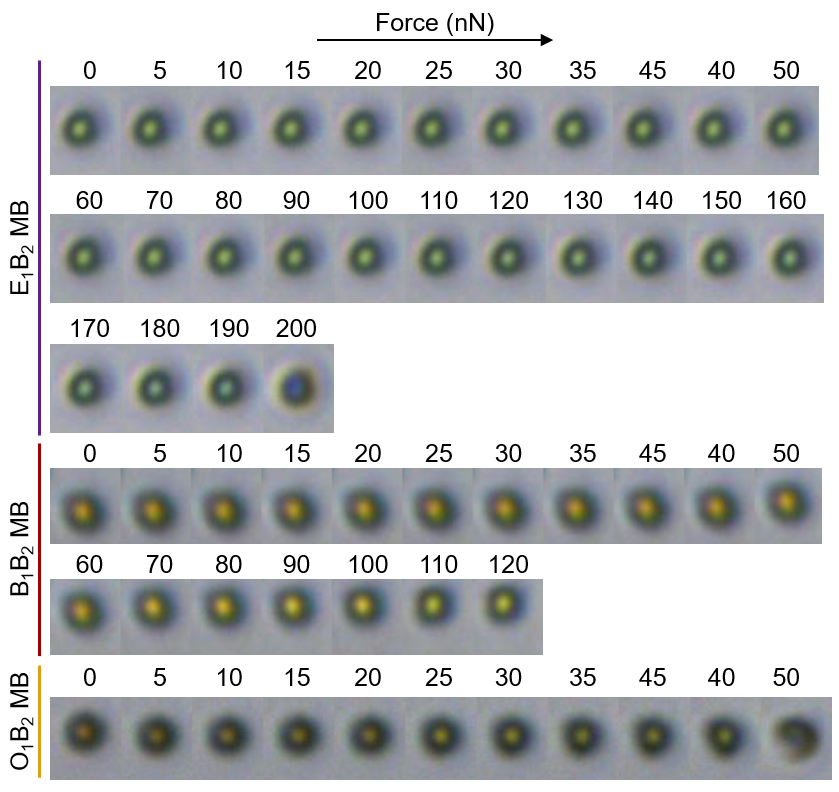  **Figure S6.** Representative optical micrographs of individual MB from each sample under progressively increasing force applied by a cantilever until MB collapse. Each square corresponds to an area of 3 × 3 µm^2^. |
| --- |

| **Table S1. Drug molecules per individual MB and drug loading per 1 × 10^9^ MB.** | | |  |
| --- | --- | --- | --- |
| **Sample** | **Drug molecules  per individual MB** | **Drug loading  (ng / 1 × 10^9^ MB)** | |
| E_1_B_2_ MB | (0.96 ± 0.07) × 10^6^ | 56 ± 4 | |
| B_1_B_2_ MB | (1.45 ± 0.20) × 10^6^ | 85 ± 12 | |
| O_1_B_2_ MB | (2.09 ± 0.11) × 10^6^ | 122 ± 7 | |

| **Table S2. The contents of the simulation boxes for all samples.** The first number of water molecules corresponds to the system before coumarin loading, the second number corresponds to the water added to the expanded part of the box. | | | | |
| --- | --- | --- | --- | --- |
| Sample | Polymer chains | Triton X-100 molecules | Coumarin 6 molecules | Water molecules |
| E_1_B_2_ MB | 417 | 93 | 100 | 41842 + 43526 |
| B_1_B_2_ MB | 210 | 139 | 100 | 48756 + 42863 |
| O_1_B_2_ MB | 193 | 59 | 100 | 48562 + 42730 |

| 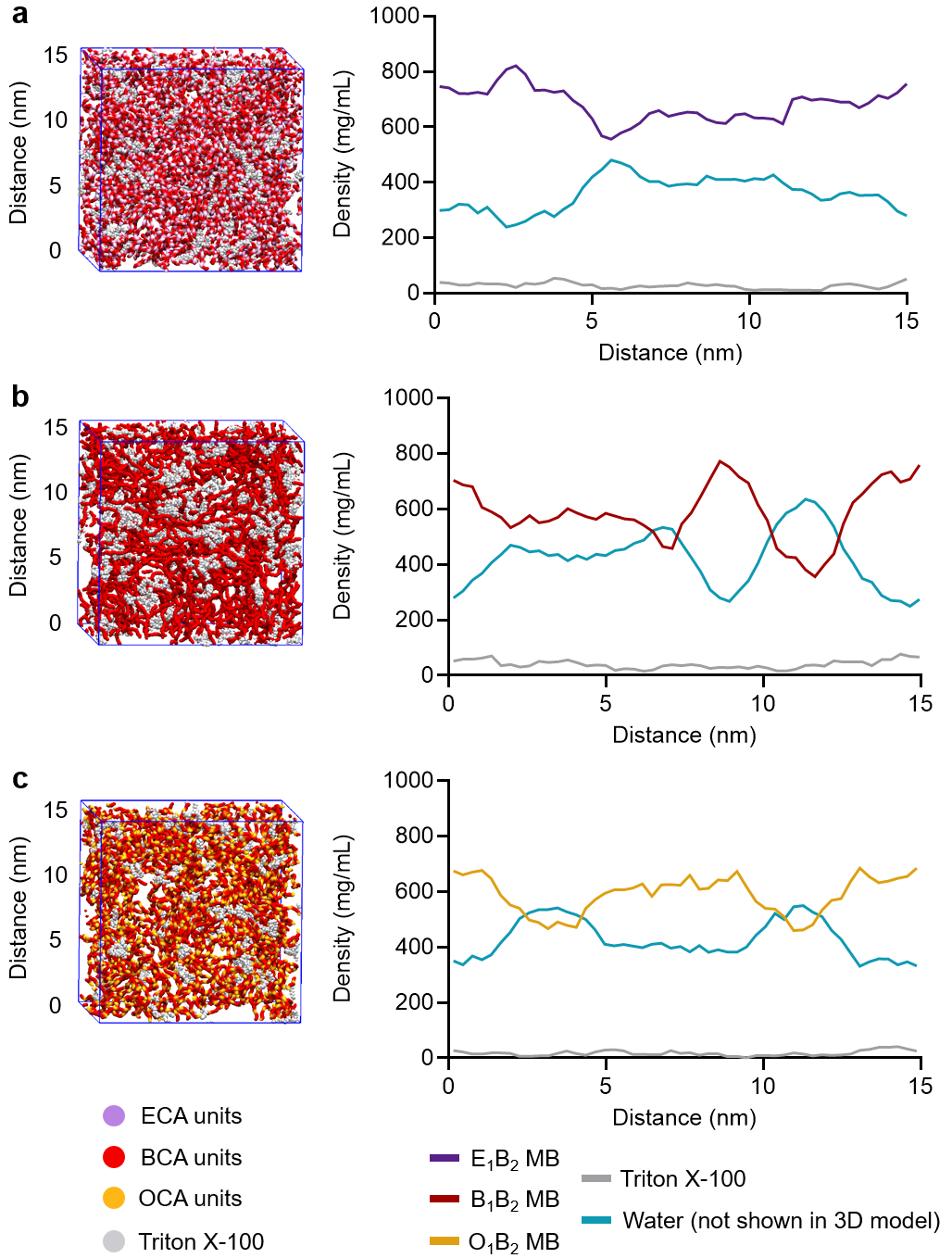 **Figure S7.** **Simulation snapshots of the polymeric MB shells and corresponding density profiles.** Simulations of the MB synthesized with different cyanoacrylate composition: (**a**) E_1_B_2_ MB, (**b**) B_1_B_2_ MB, and (**c**) O_1_B_2_ MB. In the simulation snapshots, the water molecules are not displayed for clarity, however, they were considered during the simulation, as shown in the density profiles. |
| --- |

| 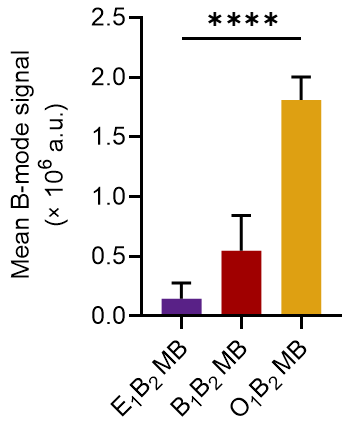 **Figure S8. Quantified mean signal intensities (arbitrary units, a.u.)** **of the polymeric MB at 4 % in B-mode.**. (****) indicates groups that are significantly different with *p* < 0.0001 (one-way ANOVA with post hoc Tukey HSD test). |
| --- |

| 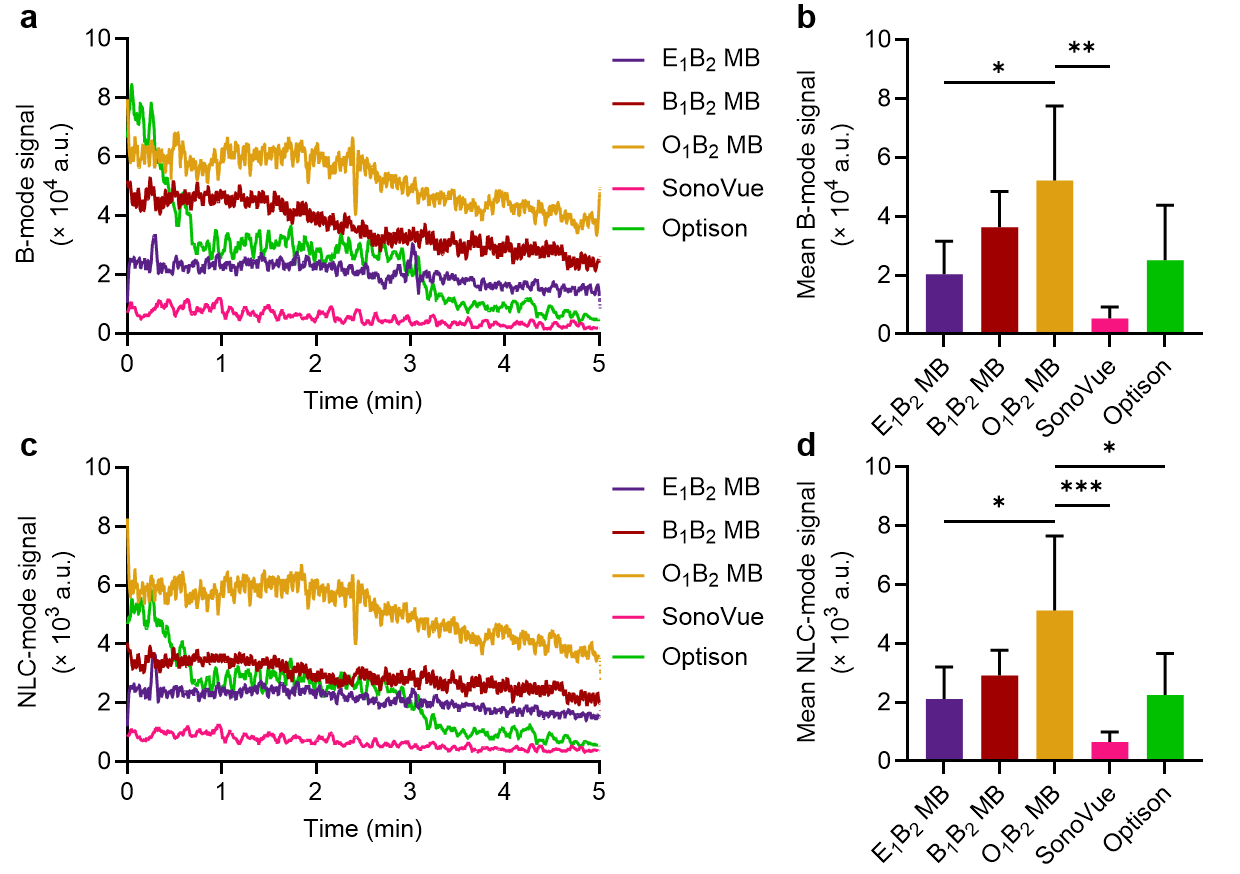 **Figure S9. Acoustic stability assessment of MB in the flow phantom**. Signal intensity (arbitrary units, a.u.) over time curves in (**a**) B-mode and (**c**) NLC-mode. Corresponding mean signal intensities are shown in (**b**) B-mode and (**d**) NLC mode. Imaging was performed at 4 % acoustic power and a center frequency of 18 MHz. SonoVue and Optison were used as representative soft-shelled and hard-shelled clinical MB formulations, respectively. Values represent mean ± standard deviation. (*), (**) and (***) indicate groups that are significantly different with p < 0.05, p < 0.01 and p < 0.001, respectively (one-way ANOVA with post hoc Tukey HSD test). |
| --- |

| 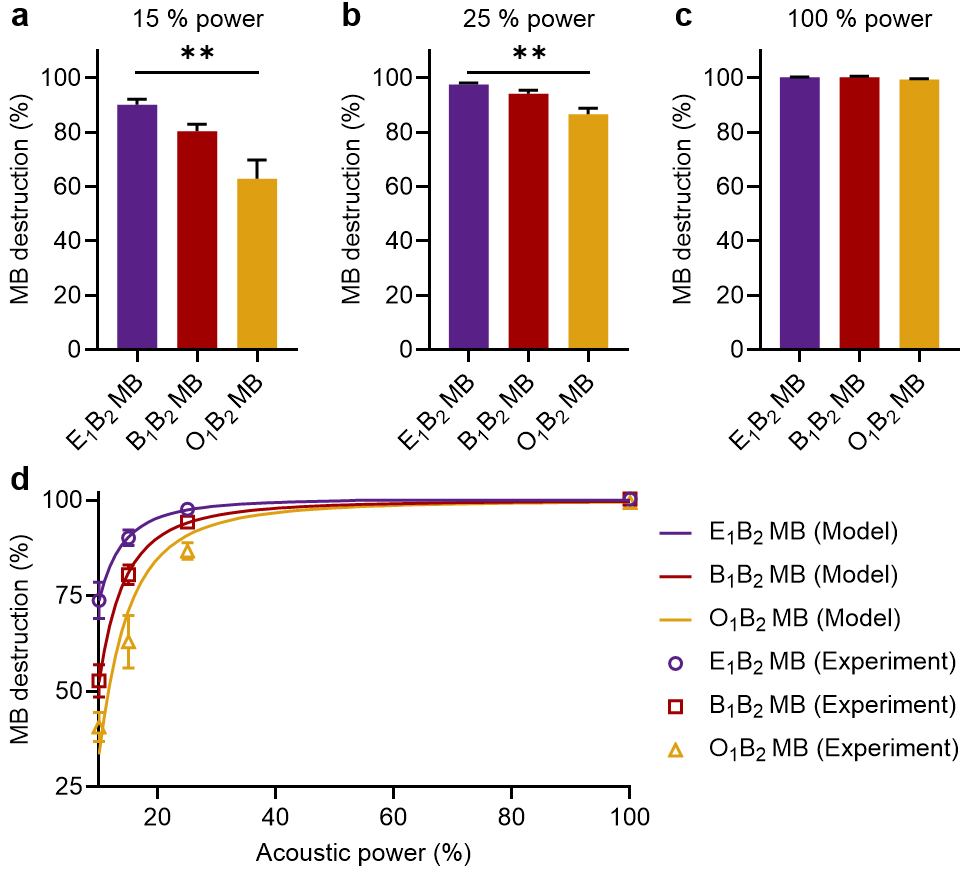  **Figure S10. Experimental and simulated destruction rates of the PACA MB.** (**a–c**) destruction rate after exposure to (**a**) 15 % power (mechanical index of 0.105), (**b**) 25 % power (mechanical index of 0.175), and (**c**) 100 % power (mechanical index of 0.7) for 5 seconds. (**d**) Prediction of the destruction ratio in the three MB systems. (**) indicates groups that are significantly different with *p* < 0.01 (one-way ANOVA with post hoc Tukey HSD test). |
| --- |

|   **Figure S11. Graphical explanation of MB destruction rate simulations.** Deformation of the system of MB with boundary *S* (left: initial state with radius *S_0_*, right: current state with radius *S*). Individual MB are visualized by dashed circles. Under US exposure, MB oscillate around their mean positions, leading to an expansion of the system boundary. |
| --- |

| 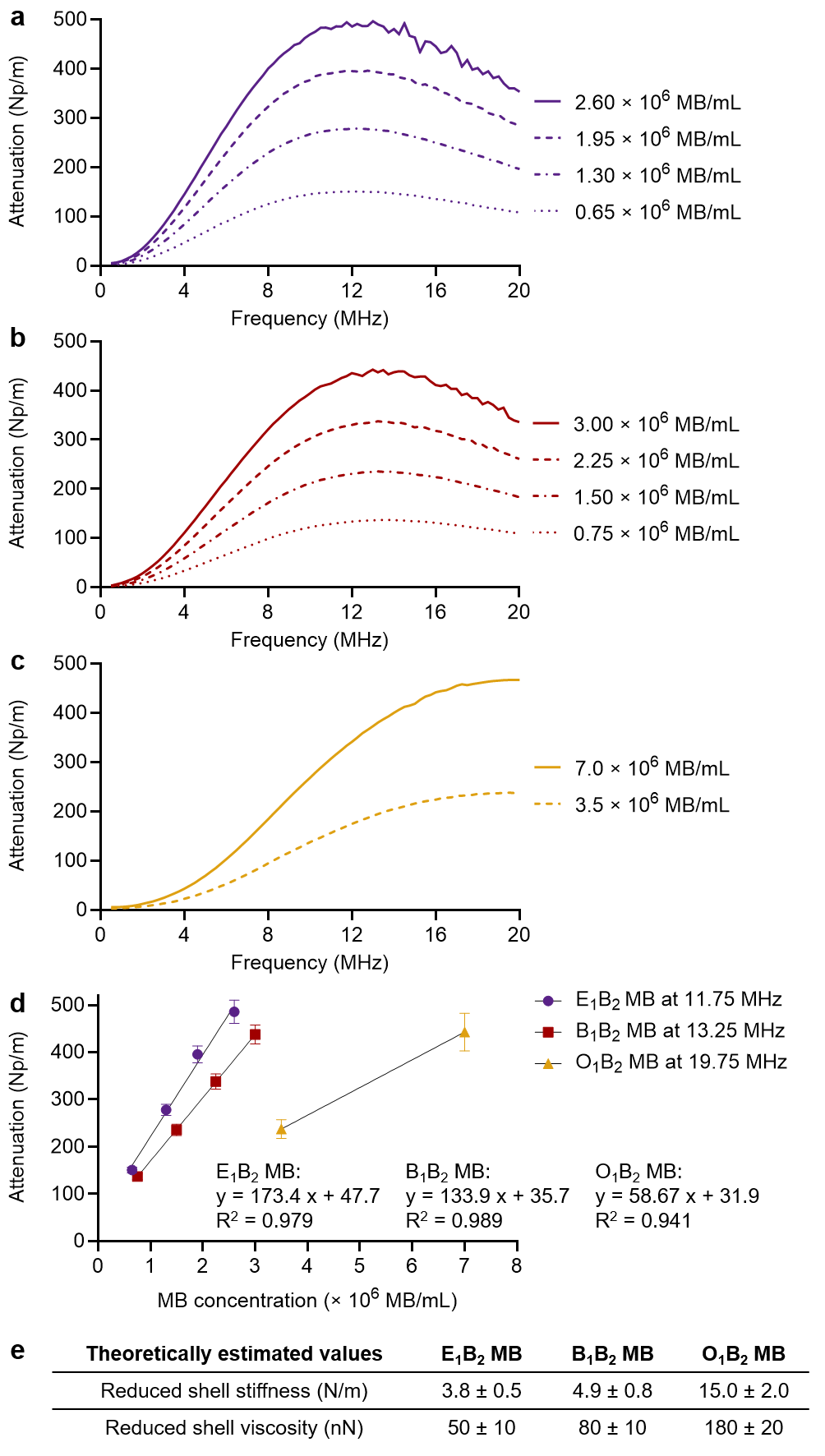 **Figure S12. Acoustic attenuation spectra of the MB at different concentrations.** (**a** – **c**) Representative spectra obtained for (**a**) E_1_B_2_ MB, (**b**) B_1_B_2_ MB, and (**c**) O_1_B_2_ MB.  (**d**) Correlation between attenuation values and MB concentration for each MB type. (**h**) Theoretically estimated reduced shell stiffness (N/m) and shell viscosity (nN) values derived from fitted attenuation data. Each sample was measured three times to ensure consistency and reproducibility of the results. |
| --- |

| 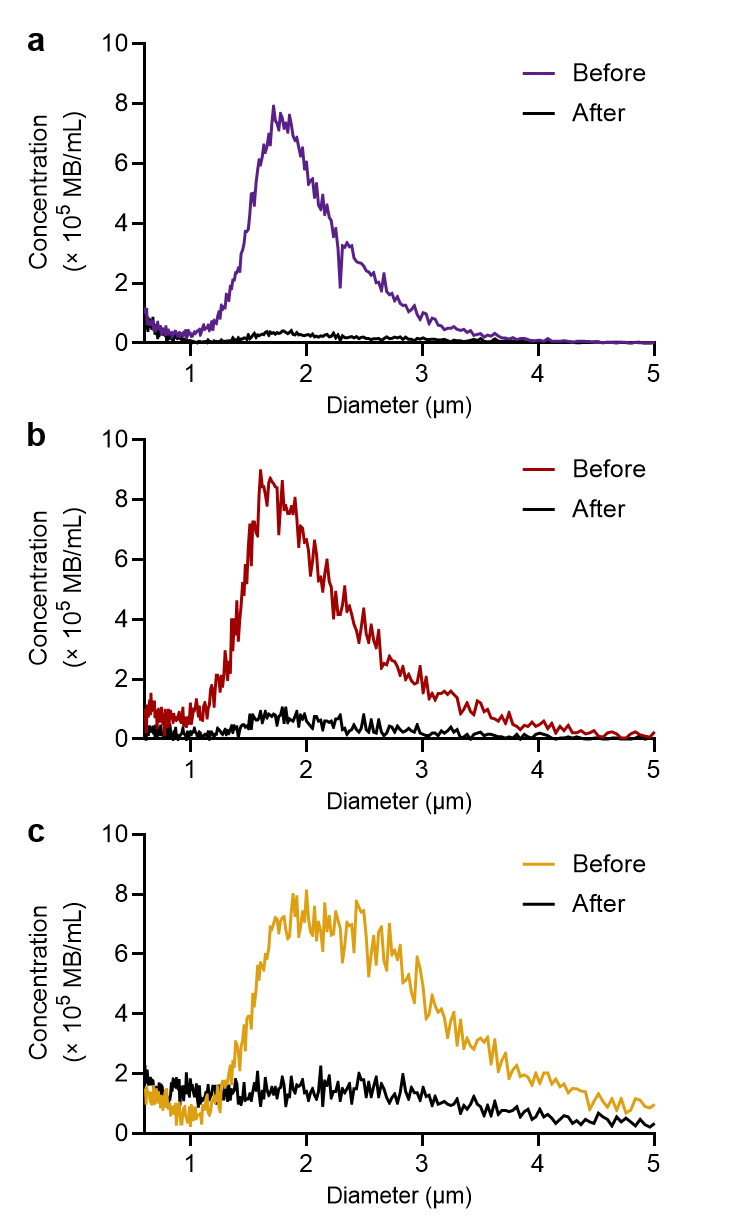 **Figure S13. Representative MB diameter distributions before and after US setup exposure for:** (**a**) E_1_B_2_ MB, (**b**) B_1_B_2_ MB, and (**c**) O_1_B_2_ MB. |
| --- |

| 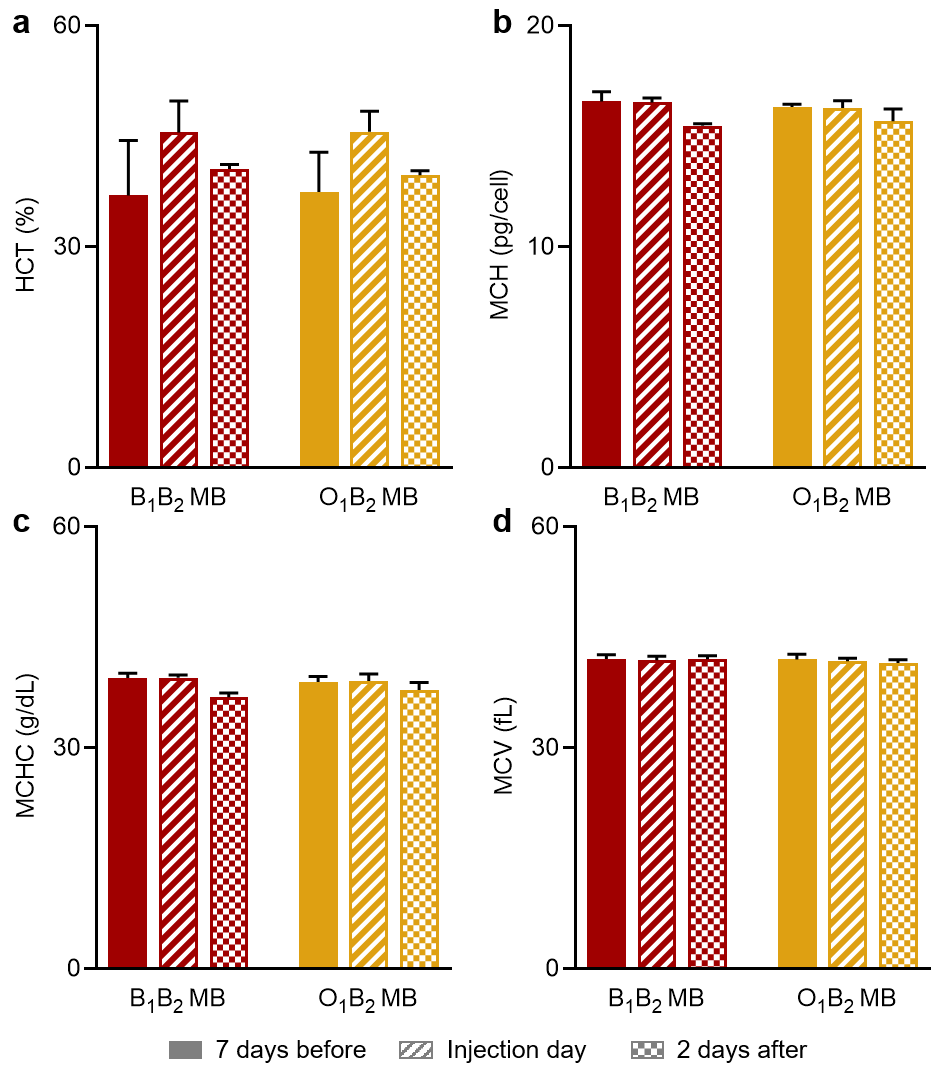 **Figure S14. Mouse blood analysis after MB administration at specified timepoints.** (**a**) Hematocrit (HCT), (**b**) mean corpuscular hemoglobin (MCH), (**c**) mean corpuscular hemoglobin concentration (MCHC), and (**d**) mean corpuscular volume (MCV) of the different groups at different time points. Values represent the mean ± standard deviation of four different animals for each group. |
| --- |
